# Neuronal cell-based high-throughput screen for enhancers of mitochondrial function reveals luteolin as a modulator of mitochondria-endoplasmic reticulum coupling

**DOI:** 10.1186/s12915-021-00979-5

**Published:** 2021-03-24

**Authors:** Luana Naia, Catarina M. Pinho, Giacomo Dentoni, Jianping Liu, Nuno Santos Leal, Duarte M. S. Ferreira, Bernadette Schreiner, Riccardo Filadi, Lígia Fão, Niamh M. C. Connolly, Pontus Forsell, Gunnar Nordvall, Makoto Shimozawa, Elisa Greotti, Emy Basso, Pierre Theurey, Anna Gioran, Alvin Joselin, Marie Arsenian-Henriksson, Per Nilsson, A. Cristina Rego, Jorge L. Ruas, David Park, Daniele Bano, Paola Pizzo, Jochen H. M. Prehn, Maria Ankarcrona

**Affiliations:** 1 Center for Alzheimer Research, Division of Neurogeriatrics, Department of Neurobiology Care Sciences and Society, Karolinska Institutet, Stockholm, Sweden; 2 Department of Medicine-Huddinge, Karolinska Institutet, Stockholm, Sweden; 3 Department of Physiology and Pharmacology, Karolinska Institutet, Stockholm, Sweden; 4 Department of Biomedical Sciences, University of Padua, Padua, Italy; 5 Neuroscience Institute, National Research Council (CNR), 35131 Padua, Italy; 6 CNC-Center for Neuroscience and Cell Biology, University of Coimbra, Coimbra, Portugal; 7 Royal College of Surgeons in Ireland, Department of Physiology & Medical Physics Department, Dublin, Ireland; 8 AlzeCure Pharma AB, Huddinge, Sweden; 9 German Center for Neurodegenerative Diseases (DZNE), Bonn, Germany; 10 Department of Clinical Neurosciences, Hotchkiss Brain Institute, Cumming School of Medicine, University of Calgary, Calgary, Canada; 11 Department of Microbiology, Tumor and Cell Biology, Karolinska Institutet, Stockholm, Sweden; 12 Faculty of Medicine, Institute of Biochemistry, University of Coimbra, Coimbra, Portugal

**Keywords:** High-throughput screen, Mitochondria, Luteolin, Mitochondria-ER contacts, Mitochondrial calcium

## Abstract

**Background:**

Mitochondrial dysfunction is a common feature of aging, neurodegeneration, and metabolic diseases. Hence, mitotherapeutics may be valuable disease modifiers for a large number of conditions. In this study, we have set up a large-scale screening platform for mitochondrial-based modulators with promising therapeutic potential.

**Results:**

Using differentiated human neuroblastoma cells, we screened 1200 FDA-approved compounds and identified 61 molecules that significantly increased cellular ATP without any cytotoxic effect. Following dose response curve-dependent selection, we identified the flavonoid luteolin as a primary hit. Further validation in neuronal models indicated that luteolin increased mitochondrial respiration in primary neurons, despite not affecting mitochondrial mass, structure, or mitochondria-derived reactive oxygen species. However, we found that luteolin increased contacts between mitochondria and endoplasmic reticulum (ER), contributing to increased mitochondrial calcium (Ca^2+^) and Ca^2+^-dependent pyruvate dehydrogenase activity. This signaling pathway likely contributed to the observed effect of luteolin on enhanced mitochondrial complexes I and II activities. Importantly, we observed that increased mitochondrial functions were dependent on the activity of ER Ca^2+^-releasing channels inositol 1,4,5-trisphosphate receptors (IP_3_Rs) both in neurons and in isolated synaptosomes. Additionally, luteolin treatment improved mitochondrial and locomotory activities in primary neurons and *Caenorhabditis elegans* expressing an expanded polyglutamine tract of the huntingtin protein.

**Conclusion:**

We provide a new screening platform for drug discovery validated in vitro and ex vivo. In addition, we describe a novel mechanism through which luteolin modulates mitochondrial activity in neuronal models with potential therapeutic validity for treatment of a variety of human diseases.

**Supplementary Information:**

The online version contains supplementary material available at 10.1186/s12915-021-00979-5.

## Background

Mitochondria are ubiquitous and multi-functional organelles involved in diverse metabolic processes, namely energy production of adenosine triphosphate (ATP), calcium (Ca^2+^) homeostasis, reactive oxygen species (ROS) regulation, and biomolecule synthesis [1]. Mitochondrial dysfunction has a main role in several pathologies; therefore, extensive toxicological and pharmacological studies have focused on the susceptibility of this organelle to both genetic and environmental damage. Although primary mitochondrial disorders caused by either nuclear or mitochondrial DNA gene mutations are relatively rare, acquired mitochondrial dysfunction has been implicated in several common diseases or conditions such as cardiovascular disease, cancer, diabetes and obesity, neurodegenerative disorders like Alzheimer’s (AD), Parkinson’s (PD), and Huntington’s diseases (HD) and aging [2]. These impairments include reduced mitochondrial biogenesis, mitochondrial membrane potential (ΔΨ_m_), and respiratory capacity, which together contribute to defective ATP production.

Several strategies have been attempted to boost mitochondrial ATP production including gene therapy, mitochondrial transplantation, metabolic manipulation, changes in diet and exercise, and the use of small molecules to target mitochondrial dysfunction. Despite the increase in randomized controlled clinical trials focusing on mitochondrial disorders [3], most of the drugs tested do not directly target mitochondria, affecting their function through indirect and, occasionally, unknown mechanisms and may lead to unanticipated adverse outcomes. Moreover, several drugs that revealed in preclinical studies substantial therapeutic potential in mitochondria [4, 5] showed limited beneficial outcomes in clinical trials due to rapid metabolism or low bioavailability in the brain [6, 7]. New-generation positively charged and/or high affinity compounds to mitochondrial membrane lipids have shown encouraging results [8]. One example is the mitochondria-targeted antioxidant Mito Q [mitoquinone mesylate] that proved to be efficient at restoring electron transport chain complexes activity and preventing oxidative stress-induced neuronal loss in spinocerebellar ataxia type 1 and inherited amyotrophic lateral sclerosis (ALS) models [9, 10]. Nevertheless, fewer than a quarter of all current mitochondria-targeting clinical trials are using new compounds [11]. Therefore, repurposing drugs that have been already approved for human use and having previously unrecognized effects on mitochondrial function could be used as an alternative strategy.

Here, we have developed a high-throughput screening (HTS) approach to select candidate compounds with potential ATP-enhancing properties in neuronal-like cells. We identified the flavonoid luteolin to be effective in increasing ATP levels in our neuronal-like cell model, which was confirmed in primary neurons and in isolated brain synaptosomes under physiological conditions. Furthermore, luteolin increased mitochondrial oxygen consumption in neurons isolated from a HD mouse model, and locomotor activity in a *Caenorhabditis elegans* (*C. elegans*) strain expressing YFP-tagged polyglutamine tract that tends to become insoluble during aging. Moreover, we uncover a new mechanism of action of this flavonoid in which luteolin-induced mitochondrial respiration and ATP production was dependent on the ER Ca^2+^-releasing channels inositol 1,4,5-trisphosphate receptors (IP_3_Rs) at specialized mitochondria-endoplasmic reticulum (ER) contact sites (MERCS).

## Results

### Differential energy sources modulate the mitochondrial energetic profile of differentiated neuroblastoma cells

**Fig. 1:**
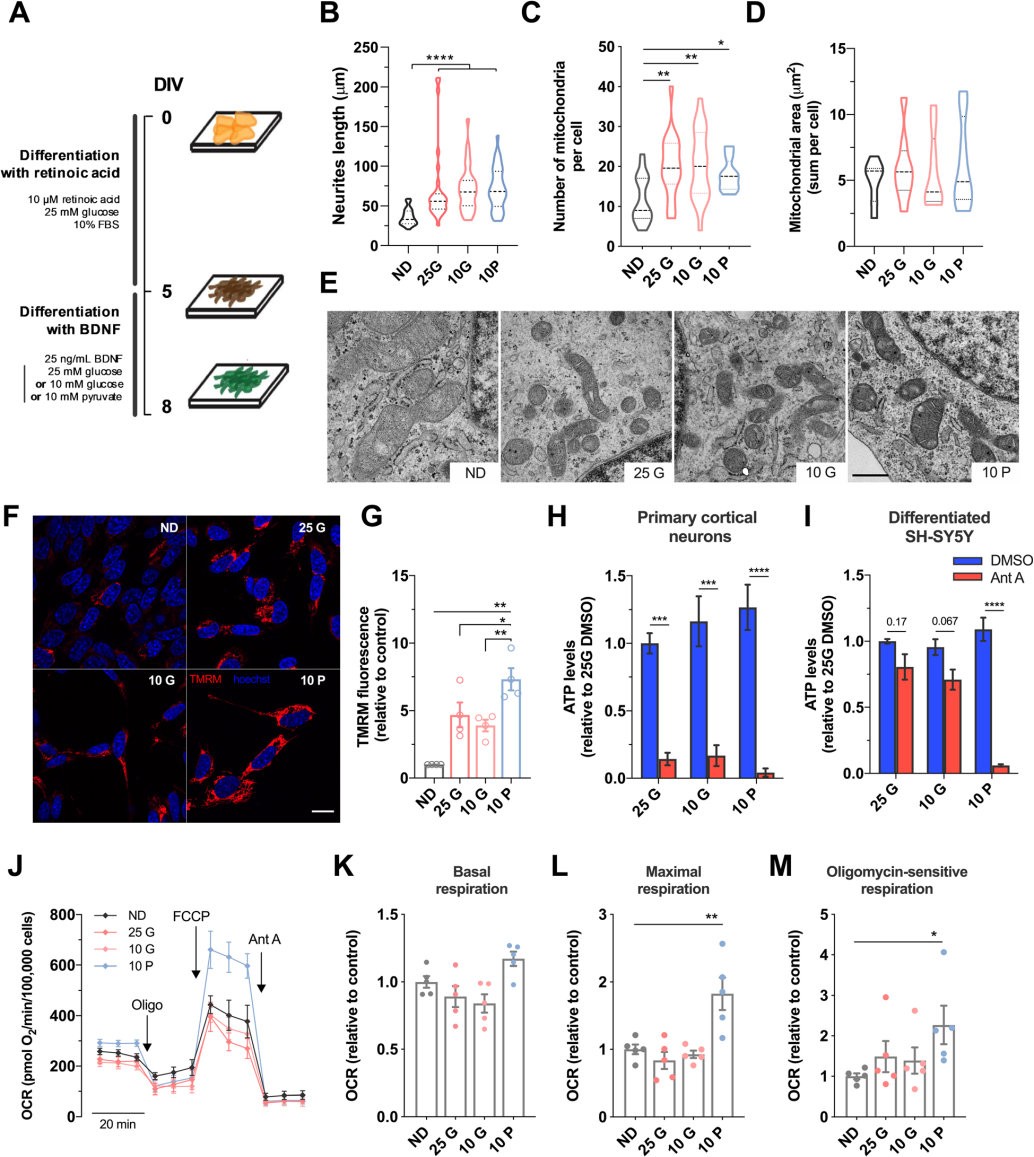
Differentiated cells grown in pyruvate show increased mitochondrial function and higher susceptibility to mitochondrial complex III inhibition. **a** Schematic representation of SH-SY5Y differentiation protocol. SH-SY5Y cells were cultured for five days in high glucose media (25 mM) supplemented with 10% FBS and 10 μM RA, followed by 3 days culture in media devoid of FBS but supplemented with 25 ng/mL BDNF and either with 25 mM or 10 mM glucose or 10 mM pyruvate. **b** Neurite length was quantified using ImageJ in SH-SY5Y cells differentiated for 8 days (*n* = 28 ND, *n* = 32 25 G, *n* = 30 10 G, *n* = 34 10 P from 4 independent experiments). **c–e** SH-SY5Y cells were fixed and imaged by TEM. Number of mitochondria profile per cell and mitochondrial profile area (in μm^2^) are represented (*n* = 20 for all conditions from 4 independent experiments). Scale bar = 1 μm. **f**, **g** ΔΨ_m_ was measured using TMRM fluorescent probe in non-quenching conditions (5 nM) in a confocal microscope (**f**) and total fluorescence was quantified (**g**) by ImageJ (4 independent experiments). Scale bar: 15 μm. **h**, **i** Total levels of ATP were measured by luminescence in differentiated SH-SY5Y or mice primary cortical neurons (as indicated) in the presence or absence of mitochondrial complex III inhibitor Ant A (3.6 μM, 30 min) (4 independent experiments run in triplicates). **j–m** OCR was quantified using Seahorse flux analyzer and basal, maximal, and oligomycin-sensitive respiration were calculated after the sequential injection of oligomycin (1 μM), FCCP (1 μM), and Ant A (0.5 μM), respectively (5 independent experiments run in quadruplicates). ND = non-differentiated; 25 G = 25 mM glucose; 10 G = 10 mM glucose; 10 P = 10 mM pyruvate. Statistical significance: ^*^p < 0.05, ^**^p < 0.01, ^***^p < 0.001, ^****^p < 0.0001 using non-parametric Kruskal-Wallis test or two-way ANOVA followed by Sidak’s multiple comparison test (in H and I)

To establish a robust and reproducible cell model for screening compounds with ATP-enhancing properties, we firstly investigated the similarities between the metabolic profile of a differentiated cell line compared to primary cortical neurons. Human neuroblastoma SH-SY5Y cells are a widely used neuronal model for which several differentiation protocols have been developed to increase their similarities with mature neurons [12–14]. We differentiated human neuroblastoma cells using an adapted two-step differentiation protocol that would favor oxidative phosphorylation (OxPHOS)-mediated ATP production. As a first step of differentiation, we exposed cells to 10 μM retinoic acid (RA) and 10% FBS, as previously described [13]. In the second step of differentiation, FBS-free DMEM media with 25 ng/mL brain-derived neurotrophic factor (BDNF) was used with either glucose (25 mM or 10 mM) or pyruvate (10 mM) (Fig. 1a). Neuronal markers were evaluated under these three differentiation conditions and compared to undifferentiated cells. The neuronal-specific protein NeuN, the presynaptic protein synaptophysin, and postsynaptic density protein 95 (PSD95) showed the same expression pattern independently of cells’ differentiation state (Additional file 1: Figure S1A). Importantly, differentiated SH-SY5Y cells expressed neuronal-encoded microtubule-associated protein 2 (MAP2) and the synaptosome-associated protein 25 (SNAP25) (Additional file 1: Figure S1A), a component of the SNARE complex with a crucial role for synaptic vesicle exocytosis [15]. Morphological light-microscopy analysis showed that the two-stage differentiation process increased cellular perimeter, while cellular area remained unaffected (Additional file 1: Figure S1B-D), most likely due to increased cellular polarity with lengthy and complex neurites (*p* < 0.0001) (Fig. 1b). Regardless of the energy source used, neither neuronal marker expression nor cellular structure was altered (Fig. 1b, Additional file 1: Figure S1A-D).

Mitochondrial ultrastructure was analyzed using transmission electron microscopy (TEM). Upon differentiation, SH-SY5Y cells showed a 1.5- to 1.8-fold increase in the number of mitochondria per cell profile (Fig. 1c); however, no significant changes were observed in total mitochondrial area (Fig. 1d). This observation, together with decreased mitochondrial perimeter of individual mitochondrion (Additional file 1: Figure S1E), may suggest that changes in mitochondria dynamics occur during the differentiation process, as previously described [16], rather than alterations in mitochondrial mass. We assessed the expression of transcription factors involved in mitochondrial biogenesis, such as nuclear-derived transcription factors NRF1 and 2, peroxisome proliferator-activated receptor gamma coactivator 1-alpha (PGC-1α) and mitochondrial transcription factor TFAM, as well as genes encoding for mitochondrial complex IV subunit Cox5b and the uncoupling protein-2 (UCP2). Although an increment of NRF2 expression was observed in all three differentiated conditions, no changes were observed in the upstream nor downstream pathways (PGC-1α and TFAM-dependent, respectively) involved in mitochondria biogenesis and transcription (Additional file 1: Figure S1F), corroborating previous observations (Fig. 1d). To determine whether differentiation influences mitochondrial bioenergetics, we used the positively charged fluorescent probe TMRM, which accumulates in the negatively charged mitochondrial matrix in non-quenching conditions (Fig. 1f). Remarkably, SH-SY5Y cells differentiated in pyruvate-enriched media (10 P) showed increased TMRM accumulation in mitochondria, which indicates increased ΔΨ_m_ compared to either undifferentiated cells (*p* < 0.01) or cells differentiated in glucose-enriched medium (*p* < 0.01 vs 10 G, *p* < 0.05 vs 25 G) (Fig. 1f, g). Using a luciferase-based assay for cellular ATP levels, we further compared differentiated SH-SY5Y cells and primary cortical neurons. As expected, mature neurons showed high susceptibility to mitochondrial complex III inhibitor antimycin A (Ant A), independently of carbon source present in growth media, which completely depleted cellular ATP pool (*p* < 0.001 or *p* < 0.0001) (Fig. 1h). In contrast, differentiated SH-SY5Y grown in 25 mM glucose (25 G) or 10 mM glucose (10 G) showed no vulnerability to Ant A treatment. Importantly, differentiated SH-SY5Y grown in 10 mM pyruvate (10 P) showed a similar energetic profile to primary cortical neurons, with an approximate 90% decrease in the ATP pool upon Ant A treatment (*p* < 0.0001) (Fig. 1i). To further confirm whether 10 P SH-SY5Y cells were suitable to assess mitochondrial function, we measured oxygen consumption rate (OCR) using Seahorse extracellular flux analysis (Fig. 1j–m). In agreement with data shown in Fig. 1g, i, 10 P SH-SY5Y cells showed increased maximal respiration (*p* < 0.01) and mitochondrial ATP synthase-driven ATP production (*p* < 0.05), relative to undifferentiated SH-SY5Y cells (Fig. 1 m).

### A cell-based HTS platform for the identification of mitochondrial enhancers

**Fig. 2:**
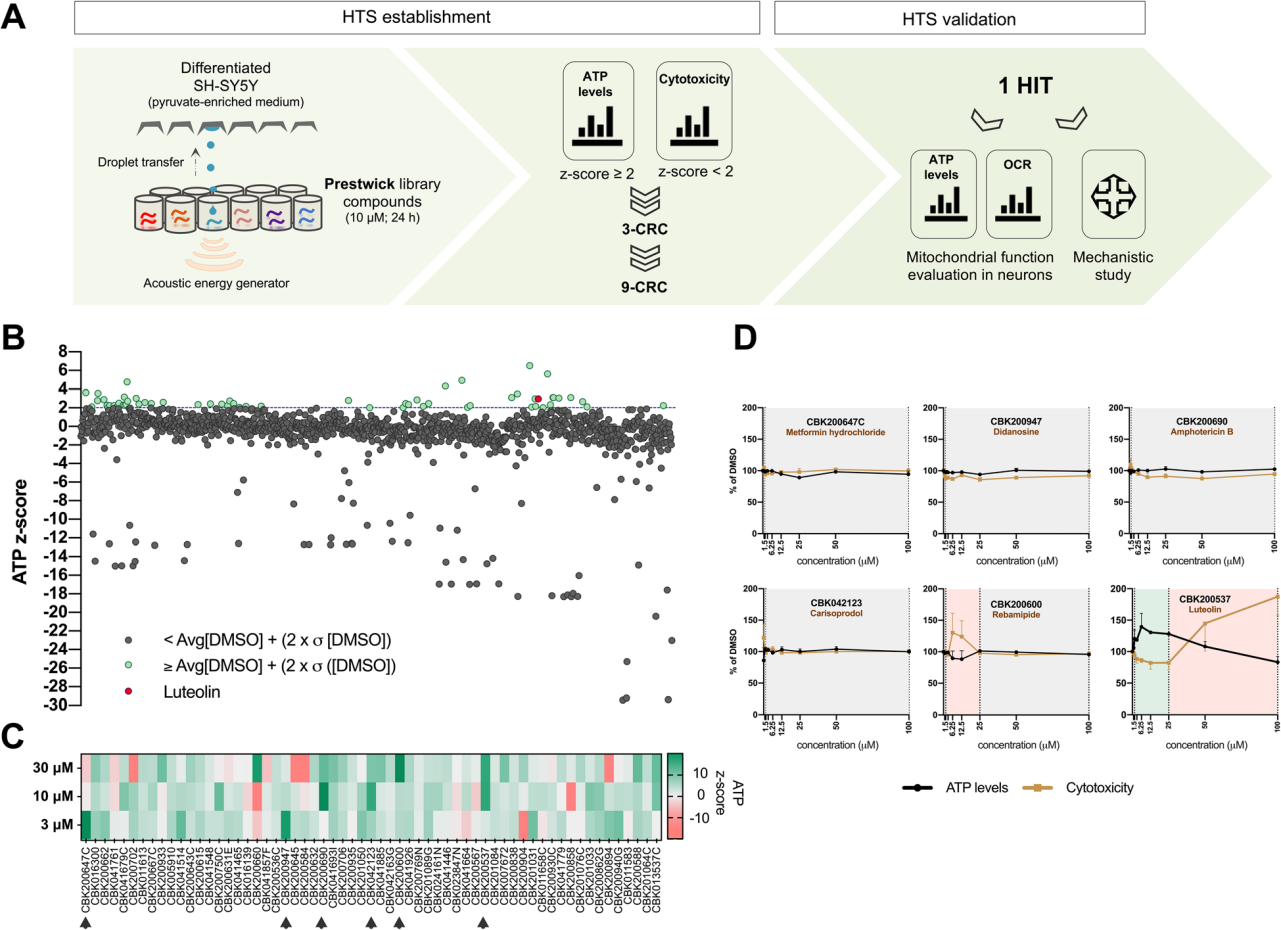
HTS reveals luteolin as a single hit to increase mitochondrial bioenergetic function. **a** Pipeline of the HTS design and validation for compounds targeting mitochondria. **b** 10 P-differentiated SH-SY5Y cells were incubated with 1200 Prestwick library-selected compounds (10 μM, 24 h), which were automatically transferred to 384-well plates using an acoustic dispenser. Total ATP levels were measured, and hits were selected when ATP *z*-scores ≥ 2 and cytotoxicity *z*-scores ≤ 2 (green dots). Luteolin is marked in red. **c** Heatmap representing the ATP *z*-scores of selected hits from B in a 3-CRC (24 h incubation). Arrow heads represent compounds with highest *z*-scores (> 6) or dose-dependent increase in ATP *z*-scores that were selected to be tested in a 9-CRC in **d**. **d** Quantification of ATP levels and cytotoxicity in a 9-CRC (24 h incubation) of the selected hits from **c**. Green background indicates range of concentrations with observed increases in ATP levels, while red background indicates increased cytotoxicity

To identify mitochondrial enhancers, we adapted pyruvate-driven differentiated 10 P SH-SY5Y cells to grow in 384-well plates suitable for automated cell plating and compound treatments. HTS was performed using the Prestwick Chemical Library, a unique collection of 1200 off-patent approved compounds (FDA, EMA and other agencies) of high chemical and pharmacological diversity. Cells were then exposed to compounds (or controls: DMSO and Ant A), dispensed at final concentration of 10 μM for 24 h. Two readouts were then quantified: the capacity of cells to generate ATP (using a luciferase-based assay) and the loss of membrane integrity, a measurement of cytotoxicity (Fig. 2a). Although total ATP levels were quantified here, these cells are expected to produce ~ 95% of their ATP through OxPHOS (Fig. 1i). All compounds that presented a *z*-score for cytotoxicity higher than 2 (i.e., higher than 2× standard deviation (σ) of DMSO above the mean of DMSO) were disregarded from further validation. This cut-off method is widely used to successfully normalize data in a plate-wise approach that was applied in this study [17]. These compounds represented 5.7% of the total library tested (Additional file 1: Figure S2A). Ant A was used as a positive control for mitochondria toxicity and presented an average decrease in ATP levels of 42.5%, while having no effect on cell viability (Additional file 1: Figure S2B, C). On the other hand, a cut-off of 2 for the ATP *z*-score was applied to identify active compounds, which generated 61 primarily hits (Fig. 2b, Additional file 2). These selected compounds underwent further validation steps, including measurements of ATP levels and cytotoxicity analysis in a 3-concentration response curve (CRC) comprising 3, 10, and 30 μM (Fig. 2c, Additional file 1: Figure S2D, Additional file 3). We found that 52 of the initial 61 selected compounds resulted in an ATP *z*-score equal or higher than 2 (white to green color gradient) for the 10 μM concentration, revealing lower false discovery rate and the high reproducibility of the HTS. From these, we selected six compounds presenting the highest *z*-scores or/and ATP levels dose-dependent increase (indicated by arrowheads), together with cytotoxicity *z*-score lower than 2 (Fig. 2c, Additional file 1: Figure S2D, Additional file 3). Interestingly, some of these validated hits such as metformin and rebamipide had been previously described to target mitochondria by modulating mitochondrial complexes activity or ROS production [18–20]. A final 9-CRC was established for the six compounds and a single hit was selected based on its robust and dose-dependent effect on ATP levels. Luteolin (3′,4′,5,7-tetrahydroxyflavone, CBK200537), a common flavone known for its antioxidant and cytoprotective properties, induced up to 40% increase in ATP levels. Importantly, decreased cytotoxicity from a range of concentrations of luteolin between 1.5 and 25 μM was observed (Fig. 2d), indicating that this increase in ATP is not detrimental and may indicate increased likelihood of reaching maximal cellular ATP levels.

### Validation of luteolin as a mitochondrial enhancer in neurons

**Fig. 3:**
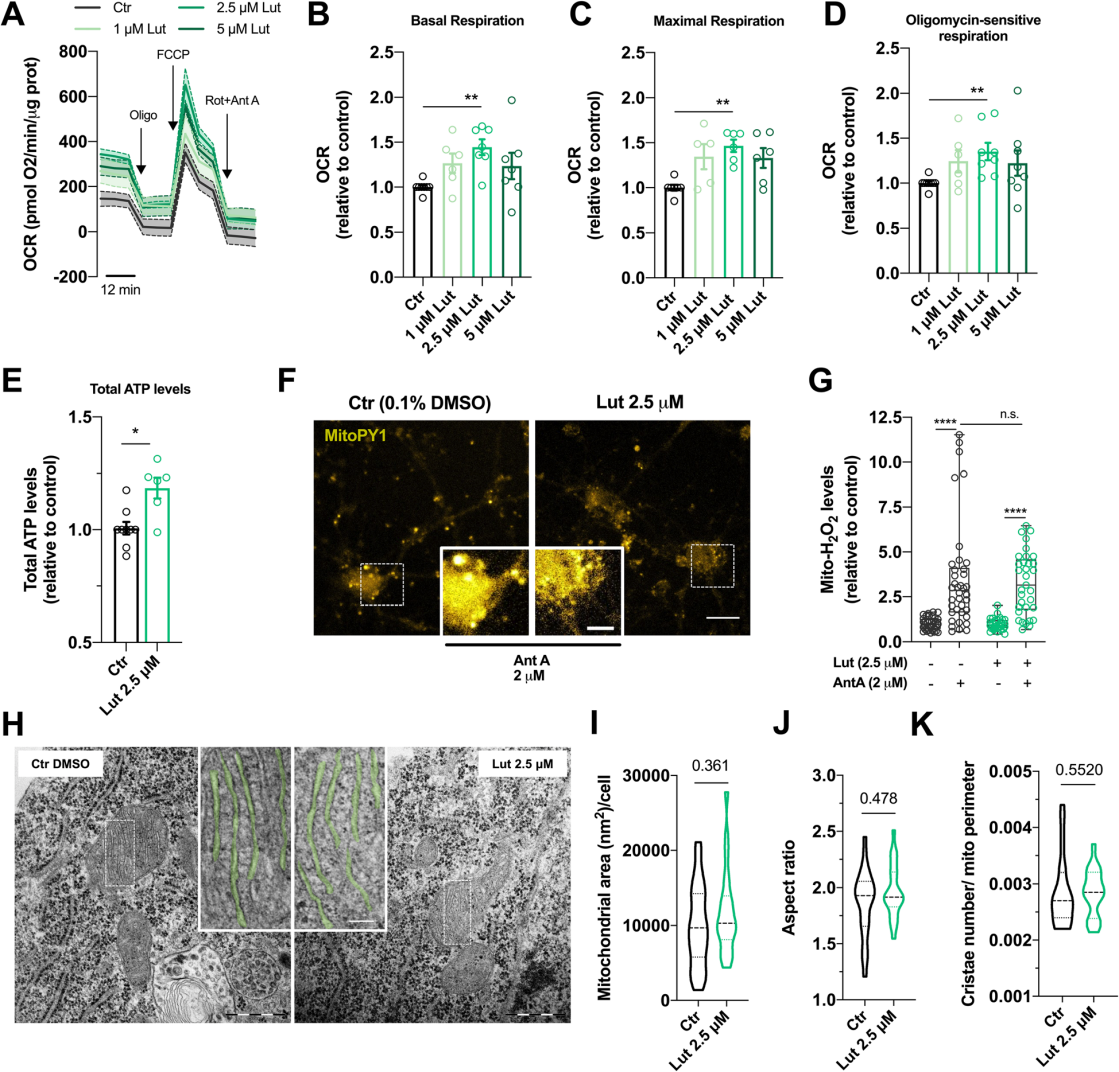
Luteolin boosts mitochondrial respiration and ATP production without affecting mitochondrial mass or structure. **a–d** Cortical neurons were treated with luteolin (Lut) for 16 h at the indicated concentrations. OCR was quantified using the Seahorse analyzer. Basal respiration, maximal respiration, and oligomycin-sensitive respiration coupled to ATP synthesis were calculated after the sequential injection of oligomycin (1 μM), FCCP (1 μM), and Ant A (0.5 μM) plus Rot (0.5 μM) (*n* = 6–7 run in triplicate; each point in the graph represents one independent experiment). **e** Cortical neurons were treated with luteolin (2.5 μM, 16 h), and total ATP levels were quantified by luminescence (*n* = 6 run in quadruplicate; each point in the graph represents one independent experiment). **f**, **g** Neurons were treated with 2.5 μM luteolin for 16 h. Mitochondrial H_2_O_2_ levels were quantified using MitoPY1 (10 μM, 25 min) in an epifluorescence cell observer microscope. After 10 min of basal reading, mitochondria were challenged with Ant A (2 μM). Representative images show MitoPY1 fluorescence before and after (insets) Ant A addition (scale bar = 25 μm; 10 μm for insert). Two-way ANOVA revealed an effect of Ant A *F*(1,137) = 65.85, *p* < 0.0001 (*n* = 37 −Lut −AntA, *n* = 38 +Lut −AntA, *n* = 33 –Lut +AntA, *n* = 33 +Lut +AntA, from 3 independent experiments). **h** Representative images of TEM in primary cortical neurons. Insets show an amplified image of mitochondrial cristae that is highlighted in green (scale bar = 500 nm; 150 nm for insert). **i–k** Mitochondria profile area (in nm^2^), aspect ratio, and cristae number per mitochondrial perimeter were quantified by TEM (in **i** and **j**, *n* = 23 Ctr, *n* = 22 Lut 2.5 μM from 3 independent experiments; in **k**, *n* = 19 from 3 independent experiments). Each *n* represents one cell. Statistical significance: ^*^p < 0.05, ^**^p < 0.01 using non-parametric Kruskal-Wallis or Mann-Whitney test (in **b–e**), ^****^p < 0.0001 using 2-way ANOVA followed by Tukey’s multiple comparisons test (in **g**); ns = non-significant

Extensive in vitro and in vivo evidence suggested luteolin as a neuroprotective agent through different mechanisms. Although luteolin role as an activator of nuclear factor erythroid 2-related factor 2 (Nrf2) pathway is well established [20, 21], the pharmacological mechanisms are largely unexplored as well as luteolin actions on mitochondrial function. To validate luteolin as a positive hit in a more robust neuronal model, we used mature mouse embryonic cortical cultures and dose-tested five concentrations of luteolin (1–20 μM) at three different time-points, based on Fig. 2d. OCR was evaluated using the Seahorse analyzer. We found that 2.5 μM luteolin induced an increase in both basal, maximal, and oligomycin-sensitive respiration after 6 h of incubation (p < 0.01) (Additional file 1: Figure S3A-D) that was maintained up to 16 h of treatment (*p* < 0.01) (Fig. 3a–d, Additional file 1: Figure S3E-H), with no significant effects on viability (Additional file 1: Figure S3P). After 24 h of luteolin incubation, the effect on mitochondria respiration was no longer detectable for all concentrations tested (Additional file 1: Figure S3I-L). These data are corroborated by luteolin’s pharmacokinetic properties, establishing luteolin mean retention time at ~ 18 h and elimination half-life (*t*_1/2_) at 7.5 h [22]. Additionally, luteolin effects on OCR showed a bell-shape concentration response curve, since higher concentrations (10 and 20 μM) did not result in significant changes in mitochondrial respiration (Additional file 1: Figure S3B-D, F-H). These results are in accordance with previous published data indicating that higher luteolin concentration leads to activation of mitochondria-associated apoptotic mechanisms in cancer cells while maintaining its antioxidant properties [23]. Total ATP levels were also evaluated in neurons by luciferase-based assay as previously performed in differentiated 10 P SH-SY5Y cells as an accurate validation of our HTS. In line with Seahorse experiments, luteolin significantly increased ATP levels by 20% (*p* < 0.05) (Fig. 3e), a similar increase observed in differentiated 10 P SH-SY5Y in the same concentration range. Despite increased respiration and ATP production, ΔΨ_m_ was not affected by luteolin in any concentrations nor time-points we tested (Additional file 1: Figure S3M-O).

As previously mentioned, luteolin acts as a ROS scavenger through a nuclear-dependent pathway by activating Nrf2. ROS are mainly produced by mitochondria and increased respiration enhances transport of electrons via the electron transport chain to prompt ROS generation. To test whether luteolin could have a direct effect on mitochondrial ROS production, we used the MitoPY1 fluorescent probe that measures mitochondrial local fluxes of hydrogen peroxide (H_2_O_2_) [24]. Both control and luteolin-treated cells stimulated with Ant A showed a significant increase in mitochondrial-driven H_2_O_2_ levels (*p* < 0.0001) (Fig. 3f, g). However, luteolin by itself or co-treated with Ant A did not affect H_2_O_2_ levels, compared to non-treated conditions, indicating that the luteolin-induced increase in mitochondrial respiration does not lead to the accumulation of mitochondrial ROS. This data also suggests that luteolin does not improve bioenergetics in neurons by scavenging ROS, as previously suggested for other cell models [20, 21].

Overall, these data validate luteolin as a mitochondrial enhancer in neuronal models and indicate that differentiated 10 P SH-SY5Y cells can exhibit similar response patterns to drug testing as mature primary neurons, and therefore can be used to facilitate drug discovery targeting the central nervous system.

### Luteolin does not affect mitochondrial area, structure, or nuclear-dependent biogenesis

We further sought to assess how luteolin exerts its positive effects on mitochondria physiology. A previous study has reported that dietary luteolin supplementation increases respiratory exchange ratio in mouse adipocytes accompanied by the upregulation of thermogenic genes due to activated 5′ AMP-activated protein kinase (AMPK)/PGC-1α [25], an energy sensing network that controls mitochondrial biogenesis and energy expenditure. To elucidate whether luteolin-triggered ATP production in neurons was related to mitochondrial biogenesis, we evaluated mitochondrial area, network, and levels of proteins regulating nuclear- and mitochondrial-dependent expression of the mitochondrial proteome. Electron micrographs of neuronal cultures were analyzed and no changes in mitochondrial profile area (Fig. 3h, i) nor in the aspect ratio (Fig. 3h, j), a geometric indicator of mitochondria elongation [26], were observed after luteolin treatment. Levels of phosphorylated AMPK, PGC-1α, and its downstream target mitochondrial DNA packaging and transcription factor TFAM [27] were also unaffected by luteolin (Additional file 1: Figure S4A). The same results were observed for several selected OxPHOS subunits (Additional file 1: Figure S4B). Since mitochondrial cristae organization greatly influences the efficiency of electron transport through mitochondrial respiratory complexes [28], we also evaluated cristae structure and crista junction proteins. Luteolin treatment affected neither cristae number (Fig. 3h, k), length and width (Additional file 1: Figure S4C, D), nor levels of Opa1 and Mitofilin (Additional file 1: Figure S4E), known to control cristae junctions.

### Luteolin-induced ATP production is dependent on IP_3_R Ca^2+^releasing channel at mitochondria-ER contact sites

**Fig. 4:**
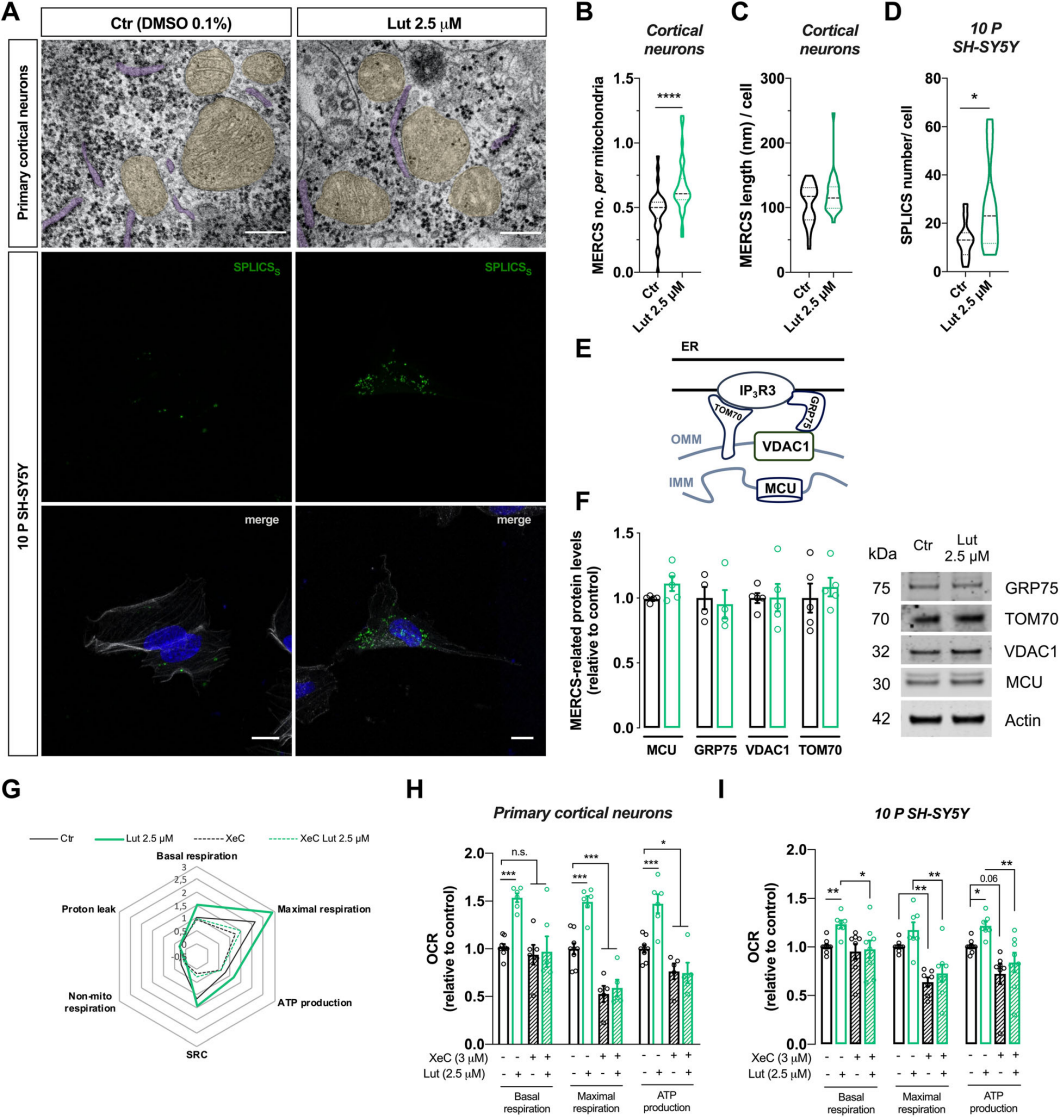
Mitochondria-ER contacts are increased by luteolin regulating mitochondrial respiration in neuronal models. **a** Representative TEM images from primary neurons evidence mitochondria (in yellow) in close contact with ER (in purple) (scale bar = 80 nm) (upper panels). Representative confocal images of differentiated 10 P SH-SY5Y cells transfected with SPLICS_S_ (in green), and f-actin labeled with phalloidin (gray) (middle and lower panels) (scale bar = 15 μm). **b, c** Number of MERCS per mitochondria profile and MERCS length were quantified by TEM (*n* = 31 Ctr, *n* = 32 Lut 2.5 μM from 4 independent experiments). **d** Differentiated 10 P SH-SY5Y cells were transfected with SPLICS_S_ and treated with luteolin for 16 h, and the number of green dots quantified. Each dot indicates a contact between mitochondria and ER (*n* = 15 Ctr, *n* = 18 Lut 2.5 μM from 4 independent experiments). **e**, **f** Schematic representation of proteins in MERCS (in **e**). MERCS proteins were quantified by western blotting using specific antibodies. Proteins were extracted from neurons treated for 16 h with DMSO or luteolin (in **f**) (*n* = 4–5; each point in the graph represents one independent experiment). OMM: outer mitochondrial membrane; IMM: inner mitochondrial membrane. **g–i** Cortical neurons (in **g**, **h**) or differentiated 10 P SH-SY5Y cells (in **i**) were treated with DMSO or luteolin (2.5 μM) for 24 h and incubated, when indicated, with XeC (3 μM) for 30 min. OCR was measured using the Seahorse flux analyzer. Spider chart lines represent the fold increase in OCR considering basal respiration of the control cells equal to 1 (*n* = 6–8 run in quadruplicate; each point in the graph represents one independent experiment). SRC: spare respiratory capacity. Statistical significance: ^*^p < 0.05, ^****^p < 0.0001 using non-parametric Mann-Whitney test (in **b**, **d**); ^*^p < 0.05, ^**^p < 0.01, ^***^p < 0.001 using non-parametric Kruskal-Wallis (in **h**, **i**)

We next questioned whether luteolin could affect the contacts between mitochondria and ER. MERCS support mitochondrial function through specific bidirectional transport of molecules such as Ca^2+^, lipids and ROS, and provide structural anchoring for mitochondrial fission [29]. Thus, electron micrographs obtained from neurons were further re-analyzed to evaluate MERCS. Interestingly, we found that luteolin treatment increased the average number of contacts calculated as the number of MERCS per mitochondrial profile (*p* < 0.0001), with no changes in MERCS length in primary cortical neurons (Fig. 4a–c). We further evaluated MERCS number in 10 P SH-SY5Y cells using the split-GFP-based contact site sensor (SPLICS) engineered to fluorescence when mitochondria and ER are in close proximity (8–10 nm) [30]. MERCS are very plastic structures and the distance between both organelles likely determines its cellular function. For Ca^2+^-MERCS, ≅15 nm is considered the optimal gap width [31]. Similar to primary cortical neurons, luteolin-treated 10 P SH-SY5Y cells showed an increased number of SPLICS dots (*p* < 0.05) when compared to controls (Fig. 4a, d), while no changes in cell area were observed between conditions.

Considering the importance of MERCS for Ca^2+^ homeostasis and its implications on mitochondrial functions [29], we decided to investigate whether luteolin could modulate Ca^2+^ signaling between the two organelles. The main complex implicated in Ca^2+^ transfer from ER to mitochondria at MERCS is composed by VDAC1 together with mitochondrial calcium uniporter (MCU) complex, on the mitochondrial side, and IP_3_Rs, on the ER side, which are chaperoned by other proteins such as the translocase of the mitochondrial outer membrane 70 (TOM70) and the glucose-regulated protein 75 (GRP75) [32, 33]. The expression of these proteins (depicted in Fig. 4e) was however not affected by luteolin treatment (Fig. 4f), suggesting that luteolin may affect the function or recruitment of these channels to the mitochondria-associated ER membrane (MAM) rather than their expression levels. To evaluate this possibility, we blocked IP_3_R-dependent Ca^2+^ release from the ER both in neurons and in 10 P SH-SY5Y cells pretreated with luteolin using Xestospongin C (XeC), a potent blocker of the receptor [34]. Under these conditions, the increase in mitochondrial basal and maximal respiration and ATP production induced by luteolin was completely abolished (Fig. 4g–i), suggesting that Ca^2+^ transfer from the ER to mitochondria is critical for mediating luteolin-dependent increase in respiration.

### Luteolin increases mitochondrial Ca^2+^ levels, ER to mitochondria Ca^2+^ transfer, and Krebs cycle activity

**Fig. 5:**
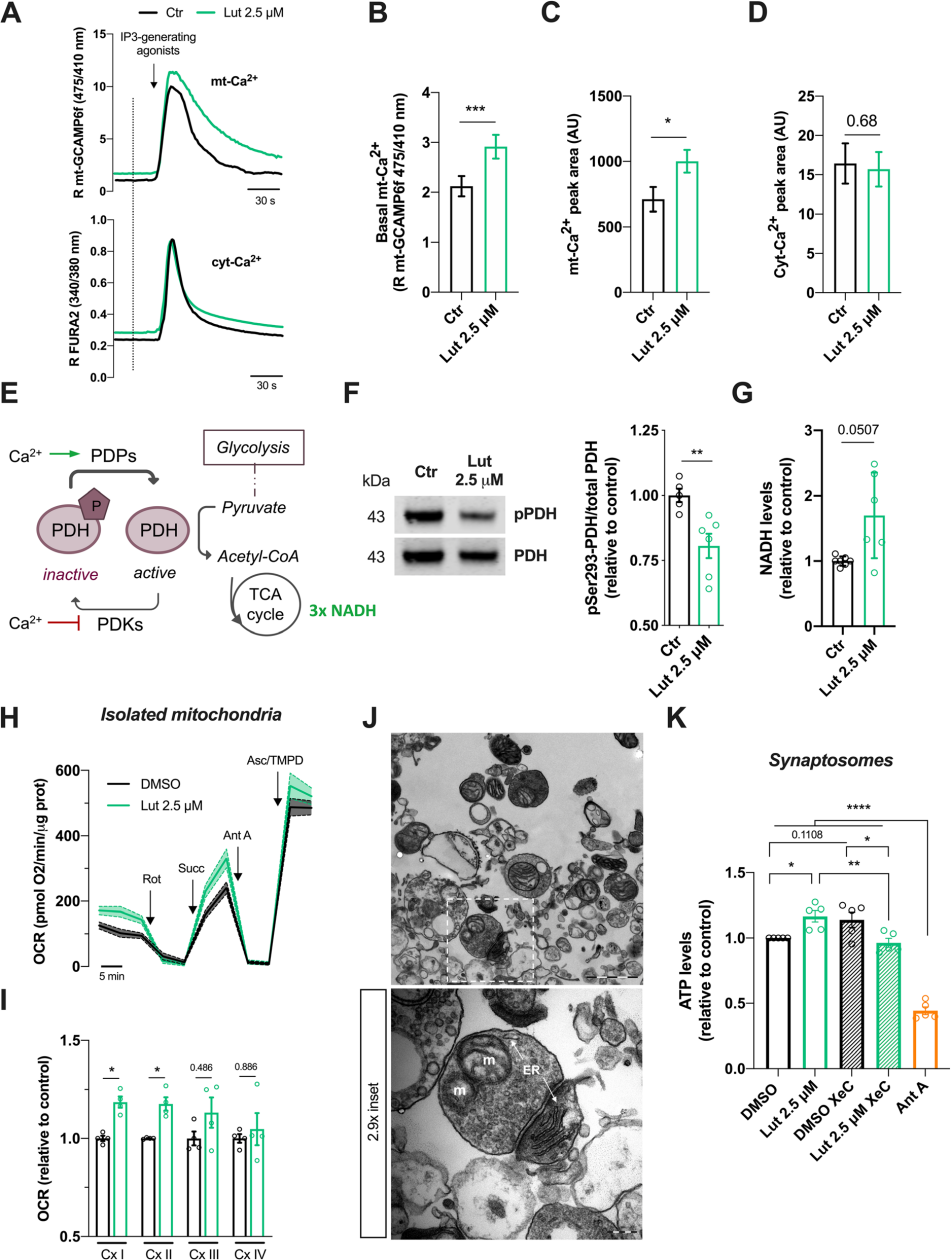
Luteolin increases mitochondrial Ca^2+^ levels, Ca^2+^ transfer from ER, and bioenergetics in synaptosomes in an IP_3_R-dependent manner. **a** Representative traces of mitochondrial (mt-Ca^2+^, upper panel) and cytosolic (cyt-Ca^2+^, lower panel) Ca^2+^ levels in cortical neurons. The arrow indicates the addition of a mix of IP_3_-generating agonists (100 μM ATP, 300 μM carbachol, and 100 μM glutamate) in a Ca^2+^-free, EGTA-containing solution. **b–d** Basal mt-Ca^2+^ levels, mean peak area of mt-Ca^2+^ and cyt-Ca^2+^ were quantified in cortical neurons treated with DMSO or luteolin (2.5 μM, 16 h) (in **b**, *n* = 121 Ctr, *n* = 147 Lut 2.5 μM; in **c**, *n* = 33 Ctr, *n* = 28 Lut 2.5 μM; in **d**, *n* = 40 Ctr, *n* = 43 Lut 2.5 μM from 3 independent cultures). **e**, **f** Schematic representation of PDH activation by calcium (in **e**). In the presence of calcium, PDH phosphatases (PDPs) are activated, favoring PDH dephosphorylation and activation. Phosphorylated and total levels of PDH E1α were quantified in neurons treated with DMSO or luteolin (2.5 μM, 16 h) by western blotting using specific antibodies (*n* = 5–6; each point in the graph represents one independent experiment). **g** NADH levels were quantified in mitochondrial-enriched extracts obtained from cortical neurons treated with DMSO or luteolin (2.5 μM, 16 h) (*n* = 6 run in triplicates; each point in the graph represents one independent experiment). **h**, **i** Electron flow was measured in cortical mitochondria isolated from WT mice using a Seahorse flux analyzer after a short 30 min incubation with luteolin or DMSO. Mitochondrial complex inhibitors or substrates, 2 μM Rot, 10 mM succinate (Succ), 4 μM Ant A, and 1 mM ascorbate (Asc)/ 100 mM TMPD, were sequentially injected to calculate mitochondrial complex I–IV activities, respectively (*n* = 4; each point in the graph represents data from one animal’s brain). **j**, **k** Pure synaptosome preparations (represented in **j**) were incubated for 30 min with luteolin (2.5 μM) and/or XeC (5 μM), and total ATP levels were quantified by luminescence. Ant A (3.2 μM, 30 min) was used as a positive control, which decreased ATP levels by 56% (*n* = 5; each point in the graph represents data from one animal’s brain). Scale bar = 1 μm (upper image); 200 nm (inset). Statistical significance: ^*^p < 0.05, ^**^p < 0.01, ^***^p < 0.001 by Mann-Whitney test when comparing two independent samples (in **b**, **c**, **f**, **i**). ^*^p < 0.05, ^**^p < 0.01, ^****^p < 0.0001 by non-parametric Kruskal-Wallis test (in **k**)

To further elucidate the role of Ca^2+^ in luteolin-induced ATP production, we evaluated mitochondrial Ca^2+^ levels using the mitochondrial Ca^2+^ probe GCaMP6f [33]. Interestingly, luteolin-treated neurons displayed increased mitochondrial Ca^2+^ levels both in basal conditions (*p* < 0.001) and upon stimulation of ER Ca^2+^ release with IP_3_-generating agonists (see “Material and methods” for detail) (*p* < 0.05) (Fig. 5a–c), revealing that increased MERCS number correlates with enhanced function. Importantly, cytosolic Ca^2+^ levels were not affected by luteolin (Fig. 5a, d), nor luteolin induced Ca^2+^ elevations per se when used as an acute stimulus (Additional file 1: Figure S5A, B).

In line with the increased mitochondrial Ca^2+^, luteolin inhibited the phosphorylation of pyruvate dehydrogenase (PDH) E1α subunit (inactive form) (*p* < 0.01), an essential enzyme that links glycolysis to the Krebs cycle (Fig. 5e, f). PDH E1α dephosphorylation is stimulated by low micromolar concentrations of Ca^2+^ ions that activate the pyruvate dehydrogenase phosphatase 1 [35]. The luteolin-induced Ca^2+^-dependent activation of PDH may in turn have accounted to the increased levels of the reduced form of nicotinamide adenine dinucleotide (NADH) (*p* = 0.0507) (Fig. 5g), a product of Krebs cycle activity and an indispensable electron carrier necessary for normal functioning of OxPHOS.

### Luteolin increases mitochondrial ATP content in isolated mitochondria and synaptosomes

To elucidate the effect of luteolin on mitochondria-ER functions, we isolated functional mitochondria containing mitochondria-associated ER membranes from mouse cortex (Additional file 1: Figure S5C) and evaluated individual respiratory complex activity using the Seahorse extracellular flux analyzer. Mitochondria were incubated with luteolin (or 0.1% DMSO) for 30 min and then fed with substrates for complex I, pyruvate, and malate, followed by sequential injections of rotenone (Rot), succinate, Ant A, and ascorbate/TMPD to induce individual stimulation or inhibition of mitochondrial complexes I, II, III, and IV, respectively, allowing the calculation of their activities [36]. Remarkably, luteolin significantly stimulated complex I and II activity (*p* < 0.05), while did not influence the two other complexes (Fig. 5h, i). These results support the idea that luteolin drives Ca^2+^-dependent PDH activation and NADH synthesis which serves as a complex I substrate. We also took advantage of a more complex ex vivo model where mitochondria and ER contacts are maintained, the synaptosome. The purity of the synaptosome preparations was evaluated by TEM where both mitochondria and ER were observed in close proximity (Fig. 5j). Mouse synaptosomes were pretreated with luteolin in the presence or absence of XeC and briefly stimulated with KCl, which allows Ca^2+^ uptake and a better filling of the ER Ca^2+^ store [37] and leads to an increase in active/total ratio of PDH and pyruvate decarboxylation [38]. As observed in vitro, luteolin significantly increased ATP levels in isolated synaptosomes (*p* < 0.05), an effect hindered by the inhibition of IP_3_Rs at the ER (Fig. 5k). These data further corroborate that luteolin works as a mitochondrial enhancer that directly targets mitochondria-ER interconnected metabolic functions.

### Luteolin protects neurons against toxicity mediated by mutant Huntingtin

**Fig. 6:**
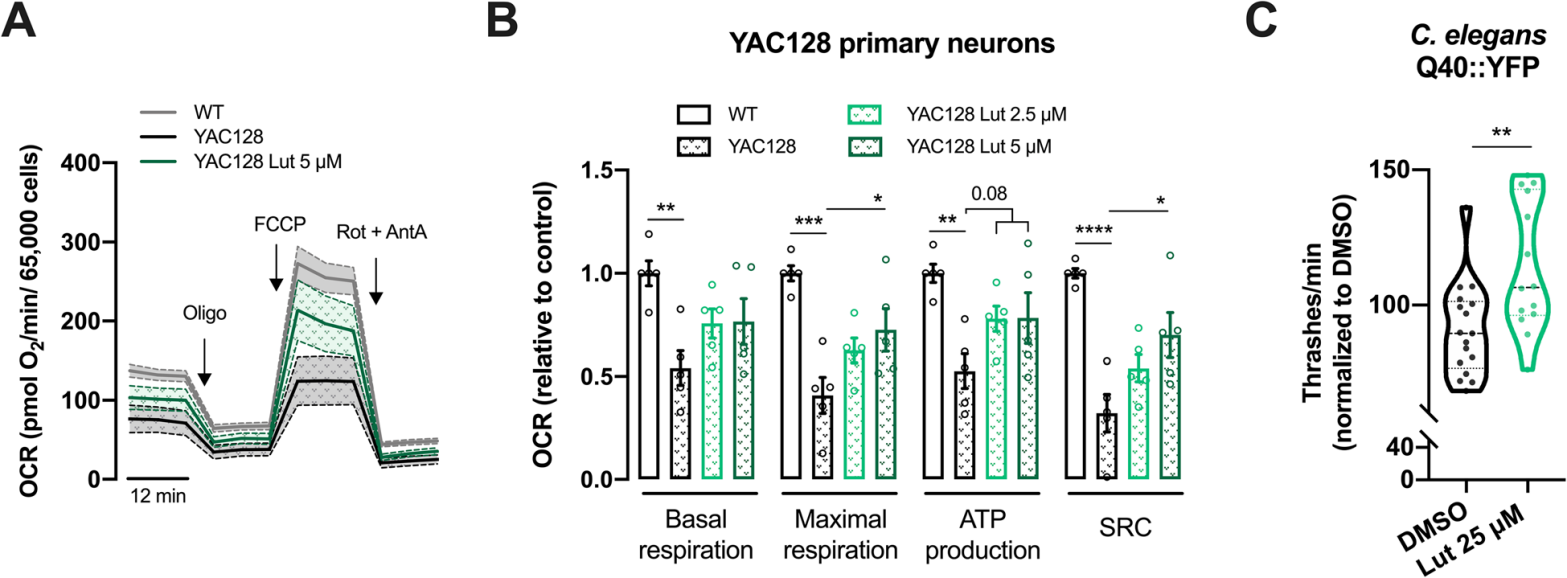
Luteolin is neuroprotective in models of HD. **a**, **b** YAC128 cortical neurons were treated with luteolin at indicated concentrations for 16 h. OCR was quantified using a Seahorse analyzer and basal respiration, maximal respiration, ATP production (oligomycin-sensitive respiration), and spare respiratory capacity (SRC) were calculated after the sequential injection of oligomycin (1 μM), FCCP (1 μM), and Ant A plus rotenone (0.5 μM) (*n* = 5 run in duplicate or triplicates; each point in the graph represents one independent culture). **c** Adult *C. elegans* carrying *rmIs110* transgene were exposed to DMSO (as a control) or luteolin (25 μM) for 96 h and trashing assay was performed (*n* = 41 from 3 biological replicates). Statistical significance: ^*^p < 0.05, ^**^p < 0.01, ^***^p < 0.001, ^****^p < 0.0001 by non-parametric Kruskal-Wallis test (in **b**); unpaired *t* test ^**^p = 0.0037 (in **c**)

Our data presented in this study reveals a potential effect of luteolin as a mitotherapeutic; therefore, we further tested this drug’s bioenergetics-modulating effect in a neurodegenerative disease model. HD is a genetic neurodegenerative disorder caused by an abnormal expansion of glutamines in the huntingtin (HTT) protein, leading to the accumulation of misfolded proteins. These abnormalities affect several cellular mechanisms including mitochondrial function, and eventually lead to neuronal cell death [39]. We isolated primary neurons from YAC128 mice embryos, a transgenic HD mouse model expressing human HTT gene containing 128 CAG repeats inserted in the yeast artificial chromosome (YAC) [40], and treated them with 2.5 and 5 μM luteolin for 16 h. OCR was evaluated using the Seahorse analyzer and severe decrease in oxygen consumption was observed in YAC128 neurons in comparison to WT neurons (Fig. 6a, b). Luteolin treatment (5 μM) partially recovered mitochondrial dysfunction in these HD neurons by significantly increasing maximal mitochondrial respiration (*p* < 0.05) and spare respiratory capacity (*p* < 0.05) by 1.8- and 2.2-fold, respectively. Additionally, the significant decrease in mitochondrial ATP levels observed in YAC128 neurons (*p* < 0.01) was reverted upon luteolin treatment (Fig. 6a, b). Interestingly, 2.5 μM luteolin showed no significant effects, suggesting that a higher drug concentration is necessary when mitochondrial function is chronically and substantially affected, compared to physiological conditions.

Finally, we tested whether luteolin could stimulate proteostasis in vivo. Thus, we employed a *C. elegans* strain expressing neuronal YFP fused to a stretch of 40 glutamines (i.e., Q40::YFP) [41]. A thrashing assay was performed in adult nematodes which were exposed for 96 h to DMSO (as a control) or luteolin (25 μM, final concentration in NGM agarose plates) (Fig. 6c). We found that luteolin exposure partially counteracted the loss of motility associated with Q40::YFP expression in neurons (Fig. 6c). This data suggests that luteolin can protect from proteotoxicity due to age-related accumulation of Q40::YFP inclusions.

## Discussion

Although it is becoming more evident that mitochondrial dysfunction is a common driving factor for a wide range of complex diseases, effective therapeutic interventions targeting mitochondria are still not readily available [11]. Previous HTSs were established to identify mitochondrial modulators or to study mitochondrial physiology in more detail. Most of these studies used cell lines derived from peripheral tissue such as mouse Hepa1-6 hepatoma cells [42] and murine myotubes [43, 44]. Only recently a HTS using primary neurons was established to identify mitotherapeutics [45]. These large-scale screenings vary in their initial readouts, which include analysis of ΔΨ_m_, mitochondrial morphology, cellular respiration, mtDNA-encoded protein synthesis, NAD(P)H levels, and ATP content [46]. Here, we established a method to differentiate human SH-SY5Y neuroblastoma cells with pyruvate-containing media during the last stage of differentiation, hence compelling them to utilize mitochondria as their main energy source, in order to match neuronal metabolism. By analyzing ATP content as a phenotypic readout of mitochondrial bioenergetics, combined with cellular viability measurements, we assured high specificity and accuracy in the identification of compounds that could increase ATP through OxPHOS with little contribution of glycolysis. The high percentage of overlap between hits selected from the primary screen and the results from the 3-CRC orthogonal screen further corroborated that our screen setup can be implemented as a powerful tool for drug discovery studies.

Our pilot screen identified 61 hits that significantly upregulated mitochondrial-dependent ATP cellular content without affecting cellular viability. Using a CRC-selection method and based on higher *z*-scores for ATP production, we confirmed luteolin as a mitochondria activity enhancer increasing ATP levels by up to 40% in the low μM concentrations range, in both differentiated SH-SY5Y cells and in primary cortical neurons. This effect was linked to increased oxygen consumption due to enhanced Ca^2+^ shuttling from the ER to mitochondria at MERCS and activity of mitochondrial respiratory complexes I and II.

Luteolin is a natural flavone compound which can be extracted from different types of plants. The pharmacological mechanisms and antioxidant characteristics of luteolin have been previously described [47], in particular its ability to increase the binding of Nrf2 to antioxidant responsive element (ARE) [20, 48, 49]. As from other screens, our HTS identified flavonoids and their derivates among the most promising compound classes to enhance mitochondrial activity. The recently published HTS using primary neurons have identified two isoflavone-derivates as mitotherapeutics. Of note, the use of ΔΨ_m_ as a single readout in the primary screen may justify why luteolin was not identified among one of the primary 135 hits [45]. Biesemann and colleagues have also identified flavanone series as novel compounds that enhanced muscle performance in aged mice by increasing intracellular ATP levels and Nrf2 gene expression [44]. More than a master regulator of the cellular redox homeostasis, Nrf2 also protects against mitochondrial toxins by modulating reduced glutathione [50] and is involved in mitochondrial biogenesis through AMPK/PGC-1α axis [51, 52]. Contrary to this evidence, no effects of luteolin on mitochondrial-derived H_2_O_2_ production at baseline or induced by mitochondrial complex III inhibition were detected here, suggesting that under our experimental paradigm mitochondria-dependent luteolin effect is not related to Nrf2 antioxidant capacity. Mitochondrial biogenesis also remained unchanged, ruling out a possible effect of luteolin through the Nrf2-AMPK/PGC-1α pathway.

Our results highlight for the first time a direct effect of luteolin on mitochondria-ER signaling platform. We observed that an acute luteolin treatment in both functional synaptosomes and isolated mitochondria containing MERCS increased mitochondria-related bioenergetic functions (e.g., ATP production and OxPHOS activity) by 20%, which was dependent on the IP_3_Rs, the ER Ca^2+^-releasing channel of the mitochondria-ER Ca^2+^ signaling platform [53]. These preparations do not contain nuclei and consist of, for the synaptosomes, a minor part of cytosol-containing synaptic vesicles and ER [54], with mitochondria being the main source of ATP production. Furthermore, we found that luteolin-dependent ATP-producing effect was related with activation of PDH that catalyzes the irreversible entry point reaction of pyruvate into the Krebs cycle, driving the production of the reduced form of electron carriers that, eventually, influenced the increased activities of mitochondrial complexes I and II observed after luteolin treatment. Nonetheless, we should also bear in mind that other signaling pathways may partially account for this increase in mitochondrial ATP levels in intact cells, as the effects of luteolin in neurons were more pronounced than in the isolated mitochondria and synaptosomes.

In recent years, luteolin has arisen as a natural compound for regulation of intracellular Ca^2+^ content. In hypothermia-induced dysfunction of cardiomyocytes, luteolin attenuated Ca^2+^ overload. This effect was linked to luteolin ability to suppress accumulation of essential regulatory proteins for cardiomyocyte Ca^2+^ handling, such as calmodulin [55]. Moreover, luteolin controls the expression, activity, and stability of the sarcoplasmic reticulum (SR)/ER Ca^2+^-ATPase 2a (SERCA2a), a key enzyme that mediates Ca^2+^ reuptake into the SR and improves Ca^2+^ transients for cardiomyocyte contractility [56]. These observations also raise questions to a possible feedback loop mechanism whereby luteolin by increasing ATP levels may influence SERCA activity. Regulation of intracellular Ca^2+^ homeostasis has a crucial role in ATP synthesis by mitochondria. Interestingly, in addition to the activation of PDH shown here, mitochondrial Ca^2+^ regulates other mitochondrial matrix enzymes such as α-ketoglutarate dehydrogenase, isocitrate dehydrogenase [35], and several mitochondrial carriers [57]. These enzymes are key players for NADH production during Krebs cycle, further feeding mitochondrial complex I activity whose function, as we have shown, was enhanced by luteolin. This hypothesis is further supported by abrogation of luteolin effects on oxygen consumption and ATP levels following the blockade of IP_3_Rs. Surprisingly, levels of different proteins involved in MERCS Ca^2+^ signaling studied here were not affected by luteolin treatment. Data obtained in synaptosomes further excluded translational regulations induced by luteolin. This may indicate that luteolin increases organelle contact formation by modulating the recruitment or stabilization of those proteins at MAM rather than affecting their levels. In fact, proteins such as Sigma-1 receptor and TOM70 were shown to function as chaperones of IP_3_Rs at MERCS and ensure proper Ca^2+^ signaling from the ER into mitochondria [33, 58].

The novel mechanism of action of luteolin on mitochondria reported here can be valuable as a pharmacological approach for several disorders where dysfunctional mitochondria-ER juxtaposition occurs, disrupting Ca^2+^ homeostasis and energy metabolism. Indeed, aberrant connection between the two organelles occurs in several neurodegenerative diseases such as ALS, AD, PD and HD . [59–63], and metabolic disorders including obesity and type 2 diabetes [64]. While synthetic linkers or genetic strategies forcing ER–mitochondria tethering have been proved to hinder deleterious phenotypes in such diseases [64, 65], our data indicates that ER–mitochondria signaling can be targeted with small molecules. In fact, very few drugs have been so far suggested to modulate MERCS function or structure; that is the case of Sigma-1 receptor agonists which have been shown to be neuroprotective in several neurodegenerative models, including in the YAC128 HD mice [66, 67]. Here, we propose luteolin as a new MERCS modulator candidate that was able to improve mitochondrial respiratory deficits in HD neurons, which display decreased mitochondria and ER juxtaposition (unpublished data).

Luteolin is brain permeable [68] and pharmacokinetic studies using HPLC-electrochemical detection found luteolin concentrations averaging 0.5 μmol/L in human serum after a single luteolin-containing extract oral dose [69]. Remarkably, these levels are in a similar concentration range as the concentrations observed to be positive for mitochondrial function in the human cell line used in this study. Concordantly, we observed that luteolin can also stimulate locomotory activity in a *C. elegans* strain expressing Q40::YFP in the nervous system. Our data are in line with previous evidence showing that luteolin may be neuroprotective and improve motor coordination in a model of spinal cord injury [70]. Moreover, luteolin was already tested in phase II clinical trials where children with autism spectrum disorders benefited from luteolin formulations by showing reduced levels of inflammatory proteins and improved sociability with no major adverse effects (ClinicalTrials.gov identifier: NCT01847521) [71, 72].

## Conclusions

Together, our data suggest that this HTS is a valuable tool to study compounds with mitotherapeutic potential. Additionally, luteolin may offer a new pharmacologic strategy for neurodegenerative disorders where mitochondria and ER coupling might contribute to disease progression. Still, the therapeutic potential of luteolin deserves further exploration regarding target identification. Our HTS validation provides the foundation for future studies on mechanisms by which luteolin influences mitochondrial function independently of nuclear signaling.

## Material and methods

### Animals and ethical permits

C57B6/J wild-type (WT) mice were housed in the Preclinical Laboratory housing facility at Karolinska Institutet, Sweden. Colonies of hemizygous YAC128 [line HD53] (IMSR Cat. no. JAX:004938, RRID:IMSR_JAX:004938) and WT mice, with FVB/N background, were housed in the Facility of the CNC, Coimbra, Portugal. All animals were kept under conditions of controlled temperature (22–23 °C) and under a 12-h light/12-h dark cycle. Food and water were available ad libitum. All experimental procedures were carried out in accordance with the guidelines of the Institutional Animal Care and Use of Committee and the European Community directive (2010/63/EU) and protocols approved by the “Regionala Etikprövningsnämnden” and “Linköpings djurförsöksetiska nämnd” (Regional Ethics Review Board, authorization no. S53–14, ID407 and 12,779/2019), by the Faculty of Medicine, University of Coimbra (authorization no. ORBEA_189_2018/11042018), and by the Italian Ministry of Health (authorization no. D2784.N.HEH, 03/07/18). For mitochondrial and synaptosome isolation, 3-month-old WT females were used.

### SH-SY5Y cell culture and differentiation

SH-SY5Y neuroblastoma cells, obtained from American Type Culture Collection (Virginia, USA) (ATCC Cat. no. CRL-2266, RRID:CVCL_0019), were grown in Dulbecco’s modified Eagle’s medium (DMEM; Thermo Fisher Cat. no. 11965092) supplemented with 10% fetal bovine serum (FBS; Thermo Fisher Cat. no. 10270098) and 10% Pen-Strep. Cells were cultured at 37 °C in a humidified 5% CO_2_ atmosphere. For differentiation, SH-SY5Y cells were grown in DMEM supplemented with 10% FBS and 10 μM RA (Sigma, Cat. no. R2625) for 5 days. At day 5, cell media was changed to DMEM deprived of glucose, pyruvate, and FBS (Thermo Fisher, Cat. no. 11966025), which was supplemented with either 25 mM glucose, 10 mM glucose or 10 mM pyruvate, and 25 ng/mL of BDNF (Prospec, Cat no. CYT-207). Cells were maintained in culture for three more days before the screening or luteolin treatments.

### Cellular structure and neurite elongation measurements

Phase contrast images were recorded at days 5 and 8 of cellular differentiation using the Evos M5000 phase contrast microscope (Thermo Fisher) with a × 20 amplification objective. The area, perimeter of the cells, and neurite length of the cells were measured using ImageJ software (NIH, USA). Five images at random were acquired at each time point for each condition. Each experiment was performed in triplicates.

### HTS and hit identification

Approximately 2000 SH-SY5Y cells per well were plated in poly-D-lysine coated 384-well plates (Corning, Cat. no. CORN3768), differentiated in DMEM containing 10% FBS and 10 μM RA and grown at 37 °C and 5% CO_2_ for 5 days. Media was changed every second day. On day 5, media was changed to DMEM deprived of glucose and FBS and supplemented with 10 mM pyruvate and 25 ng/mL BDNF for three additional days. On day 8, compounds from Prestwick library at final concentration of 10 μM and in singlets were automatically added to the cells using an Echo dispenser (Echo555 acoustic dispenser, Labcyte, CA, USA) and incubated for 24 h in a total of six plates. Cell viability and ATP levels were measured using the Mitochondrial ToxGlo Assay (Promega, Cat. no. G8000) according to the manufacturer’s instructions. Cell viability is measured by adding the first kit solution containing a quenched peptide to the cell plate well. When in the presence of necrotic proteases released from dying cells, the peptide is cleaved emitting fluorescence, which was measured using the EnVision Multimode plate reader (PerkinElmer, USA). This step was followed by the addition of the second kit solution directly to the same plate wells. This second solution contains detergents that lyse the cells, and luciferin and luciferase. These, in the presence of ATP released from the lysed cells, lead to light emission that was measured as luminescence using the EnVision Multimode plate reader (PerkinElmer, USA).

Positive compounds were defined when they led to an increase in ATP levels equal or higher than the average of ATP value of DMSO + 2× *σ* of DMSO (*z*-score ≥ 2), and to a cytotoxicity lower than the average of cytotoxicity of DMSO + 2× *σ* of DMSO (*z*-score < 2). *Z*-score was calculated as (sample − average DMSO) / *σ* DMSO. Hits identified from the primary screening were validated in a 3-CRC, in singlets, and compounds showing higher ATP *z*-scores or a concentration-dependent increase in ATP *z*-scores, together with *z*-scores < 2 for cytotoxicity, were selected for a 9-CRC run in triplicates. For the 9-CRC, compounds were tested in three plates, one singlet per plate. In all the plates used in HTS, DMSO and Ant A (3.6 μM) were used as controls and run in 16 replicates each.

### Primary neuronal culture

Primary cortical neurons were derived from 17-day-old mouse embryos generated from the offspring of crosses between WT mice (C57B6/J or FVB/N), or between hemizygous YAC128 male mice and WT females (FVB/N). For Ca^2+^ measurements, primary cultures were obtained from cortices dissected from 0- to 1-day new-born WT mice as previously described [73]. Cortices were dissected in ice-cold Hank’s balanced salt solution (HBSS; Thermo Fisher, Cat. no. 14025092). For the YAC128 cultures, dissected cortices were kept in Hibernate E medium (Thermo Fisher, Cat. no. A1247601) for approximately 4 h or until the genotyping was concluded. Dissected tissue was pooled for each genotype. Cortices were then dissociated in neurobasal medium (Thermo Fisher, Cat. no. 21103049). Suspended cells were then filtered with a 40-μm cell strainer (Corning, Cat. no. 352340) and plated into 0.1 mg/ml poly-D-lysine (Sigma-Aldrich Cat. no. P7280) coated plates at a density of 1 × 10^5^ cells/cm^2^ in neurobasal medium supplemented with B27 (Gibco, Cat. no. 17504044) and 2 mM l-glutamine (Sigma, Cat. no. G7513). Medium was changed every sixth day by replacing half of the culture medium with freshly supplemented neurobasal medium. Neurons were maintained at 37 °C in a humidified incubator with 5% CO_2_/95% air for 14–15 days. Luteolin (Tocris, Cat. no. 2874) was dissolved in DMSO (0.1%) and added to the neurons 24 h, 16 h, or 6 h before experiments as indicated in the figure legends. Luteolin stocks were stored and protected from light at − 80 °C for no longer than 6 months.

### Gene expression analysis

Total RNA was isolated using Isol-RNA Lysis Reagent (5 PRIME, Cat no. 2302700), according to the manufacturer’s instructions. Afterwards, 1 μg of RNA was treated with Amplification Grade DNase I (Life Technologies) and from that, 500 ng was used for cDNA preparation using the Applied Biosystem Reverse Transcription Kit (Life Technologies). Quantitative Real-Time PCR was performed in a ViiA 7 Real-Time PCR system thermal cycler with SYBR Green PCR Master Mix (Applied Biosystems). Analysis of gene expression was performed using the ΔΔC_T_ method and relative gene expression was normalized to TATA-binding protein (TBP) mRNA levels. Primer sequences are available upon request.

### Cellular extracts and western blotting

Total cellular protein content was extracted by lysing the cells in RIPA buffer (150 mM NaCl, 50 mM Tris pH 7.5, 1% Triton X-100, 0.5% sodium deoxycholate, 0.1% SDS) supplemented with 1:100 phosphatase and protease inhibitors (Promega). Protein concentration was determined by Pierce BCA protein assay (Thermo Fisher, Cat. no. 23225). In total, 15–20 μg of protein was separated in NuPAGE 4–12% Bis-Tris protein gels (Invitrogen, Cat no. NP0321BOX) and transferred to 0.45 μm nitrocellulose membranes (GE Healthcare, Cat. no. 10600002). After blocking with 5% bovine serum albumin (BSA)/TBS-T, blots were incubated overnight at 4 °C with the following primary antibodies diluted in 5% BSA/TBS-T: total OXPHOS cocktail (1:1000) (Abcam, Cat. no. ab110413, RRID:AB_2629281), synaptophysin (1:1000) (Enzo Lifesciences, Cat. no. ADI-VAM-SV011-D, RRID:AB_2198857), SNAP25 (1:1000) (Biolegend, Cat. no. 850301, RRID:AB_2715872), MAP2 (1:2000) (Sigma-Aldrich, Cat. no. M4403, RRID:AB_477193), NeuN (1:1000) (Merck Millipore, Cat. no. MAB377, RRID:AB_2298772), PSD95 [6G6-1C9] (1:1000) (Abcam, Cat. no. ab2723, RRID:AB_303248), AMPK (1:1000) (Cell Signaling, Cat. no. 2532, RRID:AB_330331), p-AMPK (Thr172) (1:1000) (Cell Signaling, Cat. no. 2535, RRID:AB_331250), PGC-1α AC1.3 (1:500) (Merck Millipore, Cat. no. ST1202, RRID:AB_2237237), TFAM (1:500) (Abcam, Cat. no. ab131607, RRID:AB_11154693), TOM20 (1:1000) (Santa Cruz, Cat. no. sc-11,415, RRID:AB_2207533), TOM70 (1:1000) (Santa Cruz Biotechnology, Cat. no. sc-366,282), GRP75 (1:1000) (kindly provided by Prof. Elzbieta Glaser, Stockholm University), MCU (1:1000) (Sigma-Aldrich, Cat. no. HPA016480, RRID:AB_2071893), VDAC1 (1:1000) (Abcam, Cat. no. ab14734, RRID:AB_443084), Mitofilin (1:500) (Novus Biologicals, Cat. no. NB100-1919SS, RRID:AB_921811), Opa1 (1:1000) (BD Bioscience, Cat. no. 612606, RRID:AB_399888), pSer293-PDH (1:1000) (Merck Millipore, Cat. no. ABS204, RRID:AB_11213668), PDH (1:1000) (Santa Cruz, Cat. no. sc-377,092, RRID:AB_2716767), VDAC1 (1:1000) (Abcam, Cat. no. ab14734, RRID:AB_443084), and VAPB (1:1000) (given by Professor Chris Miller). Actin (1:2500) (Sigma-Aldrich, Cat. no. A2066, RRID:AB_476693) was used as a loading control. After washing, membranes were incubated with IRDye 800CW fluorescent secondary antibodies (1:20000) (LI-COR Biosciences) for 1 h at room temperature. Membranes were developed using Odyssey CLx (LI-COR Biosciences), and quantification was performed using Image Studio software (LI-COR Biosciences).

### Transmission electron microscopy and analysis

Neurons were washed and fixed in 2.5% (V/V) glutaraldehyde in 0.1 M phosphate buffer. The ultrathin sections were prepared using Leica Ultracut UCT (Leica, Vienna, Austria) and contrasted with uranyl acetate and lead citrate. Sections were observed in a Tecnai 12 BioTWIN transmission electron microscope (FEI Company, Eindhoven, The Netherlands) at 100 kV. Digital images were acquired with a Veleta camera (Olympus Soft imaging Solutions, GmbH, Münster, Germany) at a primary magnification of × 26,500. All mitochondria from 10 different cells were imaged per condition. Number of MERCS, mitochondria profile area (sum per cell), perimeter (per mitochondrion), and aspect ratio, as well as cristae length and width, were quantified using the freehand line tool in ImageJ (NIH, USA). The number of MERCS per mitochondria was obtained by dividing number of MERCS per number of mitochondria profile. Distances ≤ 30 nm between ER and mitochondria were considered as contacts. The aspect ratio was calculated by dividing the longer axes of the mitochondria profile by the smaller axes of the mitochondria profile. Analysis of cristae number and structure were only quantified when full mitochondrial profile could be observed (same *z* plane) and with cristae completely detectable.

### SPLICS quantification

Transfection with SPLICS_S_ (short-range MERCS) plasmid, kindly provided by Dr. Tito Calì (University of Padova, Italy) [30], was performed at day 5 of differentiation with Lipofectamine 2000 transfection reagent (Thermo Fisher Cat. no. 11668019) according to the manufacturer’s instructions. After transfection, cells were maintained in 10 mM pyruvate DMEM media. Transfected 10 P SH-SY5Y cells were treated with DMSO or luteolin (2.5 μM, 16 h) at day 7 of differentiation. Cells were fixed with 4% paraformaldehyde for 15 min, permeabilized in PBS containing 0.2% Triton X-100 for 2 min and blocked for 1 h, at room temperature in 3% (w/v) BSA in PBS. Cells were then incubated for 1 h with Alexa Fluor 594 phalloidin (1:50) (Thermo Fisher, Cat. no. A12381, RRID:AB_2315633) to label F-actin. Nuclei were labeled with 4 μg/mL Hoechst 33342 for 15 min and mounted using Vectashield antifade mounting media. Z-stack images were acquired using a Plan-Apochromat/1.4NA × 63 lens on a Zeiss LSM 880 confocal microscope (Zeiss Microscopy, Germany). Z-stack images were processed, and a 3D reconstruction was performed in Fiji software as previously described [30]. A selected face of the 3D image was thresholded to create a binary image that was further used to count the number of MERCS.

### Cellular respirometry by extracellular flux analysis

Mitochondrial respiration was assessed in non-differentiated and differentiated SH-SY5Y cells and in primary cortical neurons treated as described above. Wherever indicated, cells were treated for 30 min with 3 μM xestospongin C (XeC; Tocris Cat. no. 1280). OCR was measured using the Seahorse® XF24 or XF96 analyzers (Agilent) as an indication of mitochondrial respiration. The analysis was performed in base DMEM (Sigma-Aldrich, Cat no. 5030) supplemented with 25 mM glucose, 10 mM glucose, or 10 mM pyruvate (pH 7.4) as substrate for SH-SY5Y cells; or in base DMEM supplemented with 10 mM glucose and 0.223 mM pyruvate for primary neurons (pH 7.4). The OCR was measured at baseline and followed by sequential stimulation with 1 μM oligomycin A (Sigma-Aldrich, Cat no. 75351), 1.5 μM carbonyl cyanide-4-(trifluoromethoxy)phenylhydrazone (FCCP, Sigma-Aldrich, Cat no. C2920), and 0.5 μM Ant A (Sigma-Aldrich, Cat no. A8674) plus 0.5 μM Rot (Sigma-Aldrich, Cat no. R8875). Basal respiration, maximal respiration, and oligomycin-sensitive respiration were calculated using the Seahorse XF Cell Mito Stress test report generator from Wave 2.6.1 software (Agilent). OCR measured in pmol O_2_/min was normalized to cell number or protein content.

### Assay for ATP levels

For each biological replicate, 100 μL of CellTiter-Glo reagent (Promega, Cat. no. G9681) was added to 100 μL of cell culture medium. Plates were agitated for 2 min and incubated for 10 min at room temperature (21–23 °C) before luminescence measurement. Data were normalized to protein content. For ATP measurements in Fig. 1h, culture media was replaced with neurobasal media devoid of glucose and pyruvate (Thermo Fisher, Cat. no. A2477501), which was supplemented either with 25 mM glucose, 10 mM glucose, or 10 mM pyruvate.

### Mitochondrial membrane potential measurements

SH-SY5Y cells were plated in a 35-mm glass-bottom culture dishes (MatTek Corporation) coated with 0.1 mg/mL poly-D-lysine. Cells were incubated with tetramethylrhodamine, methyl ester (TMRM; Life Technologies Cat. no. T668) in non-quenching conditions (5 nM), and nuclei were stained with 2.5 μg/mL Hoechst (Life Technologies, Cat. no. H3570) for 15 min before imaging. Images were collected in three independent experiments and fluorescence intensity was analyzed with ImageJ. In neurons, TMRM was used in quenching conditions (150 nM) and incubated in Na^+^ medium (140 mM NaCl, 5 mM KCl, 1 mM CaCl_2_, 1 mM MgCl_2_, 10 mM glucose, 10 mM HEPES, pH 7.4) for 30 min at 37 °C. Under these conditions, retention of TMRM by mitochondria was studied to estimate changes in Δψ_m_. Basal fluorescence (503 nm excitation and 525 nm emission) was recorded using the microplate reader Fluostar Galaxy (LabVision) for 5 min, followed by the addition of 2.5 μM FCCP plus 2.5 μg/mL oligomycin to produce maximal mitochondrial depolarization and mitochondrial probe release. TMRM release was calculated based on the change in fluorescence before and after addition of oligomycin/FCCP. Data were normalized to protein content.

### Mitochondrial NADH levels

NADH levels were quantified in mitochondrial-enriched fractions using the NAD/NADH quantitation colorimetric kit (Promega, Cat. no. K337-100) following the manufacturer’s instructions. Briefly, 3 × 10^6^ neurons were resuspended in ice-cold sucrose buffer (250 mM sucrose, 20 mM HEPES/KOH (pH 7.5), 100 mM KCl, 1.5 mM MgCl_2_, 1 mM EGTA, and 1 mM EDTA) and centrifuged at 1088*g* for 12 min (4 °C) to pellet the nuclei and cell debris. The supernatant was further centrifuged at 12,000*g* for 20 min (4 °C) with the resulted pellet (mitochondrial-enriched fraction) resuspended in NADH/NAD extraction buffer. The samples were frozen three times in dry ice and centrifuged at 20,800*g* for 5 min. Extracted samples were divided into two: one remained in ice (total NAD), and the other heated to 60 °C for 30 min to decompose NAD^+^ (NADH). All NAD was then converted to NADH after 5 min incubation with a NAD cycling mix enzyme. Absorbance was measured at OD 450 nm after incubating the samples in dark for 1 h, at room temperature, with a NADH developer. NADH levels were determined using a NADH standard curve. Data were normalized to protein content.

### Mitochondrial-derived hydrogen peroxide levels

Neurons were incubated with Mitochondria peroxy yellow 1 (MitoPY1) probe (10 μM; Tocris Bioscience, Cat. no. 4428) in Na^+^ medium for 25 min at 37 °C. After incubation, MitoPY1 was washed and neurons were imaged in the same experimental medium every 30 s for 20 min using a LCI PlanNeofluar/1.3NA × 20 lens on a Carl Zeiss Axio Observed Z1 inverted confocal microscope with Zen Blue software (Zeiss, Jena, Germany). Fluorescence was recorded at 503 nm excitation and enhanced emission at 528 nm [24]. After 10 min of basal recording, Ant A (2 μM) was added in the medium. Fluorescence intensity at each time point was analyzed in Fiji using the time series analyzer plugin (v 3.0) developed by Balaji J. (2007).

### Mitochondrial and cytosolic Ca^2+^ measurements

#### Cytosolic Ca^2+^ levels

Neurons plated in 18 mm coverslips were incubated with 1 μM Fura-2/AM, 0.02% pluronic F-127 and 200 μM sulfinpyrazone for 30–40 min (37 °C) in mKRB media (in mM: 140 NaCl, 2.8 KCl, 2 MgCl_2_, 10 HEPES, 2 CaCl_2_, pH 7.4 at 37 °C) supplemented with 5 mM glucose and 0.2 mM sodium pyruvate. After washing, coverslips were mounted in mKRB supplemented media and visualized on an inverted microscope (Zeiss Axiovert 100, Jena, Germany) by a × 40 ultraviolet-permeable objectives (Olympus Biosystems GmbH, Planegg, Germany). Alternated excitation wavelengths of 340 and 380 nm were obtained by a monochromator (polychrome V, TILL-Photonics). The emitted fluorescence was collected at 500–530 nm. Images were acquired every 1 s, with 200 ms exposure time by a PCO SensiCam QE (Kelheim, Germany) camera.

#### Mitochondrial Ca^2+^ levels

Cortical neurons at 11 DIV were infected with AAV9 viral particles generated for expression of mitochondria-targeted GCAMP6f under a synapsin promoter (AAV9-syn-mtGCAMP6f), essentially as previously described [74]. Ca^2+^ imaging was performed 72 h after infection in mKRB media at 37 °C (see above), on an inverted microscope (Zeiss Axiovert 100) with a × 40 oil objective in the 500–530 nm range (by using a band-pass filter, Chroma Technologies). Mitochondria-targeted GCAMP6f was sequentially excited at 475 nm (for 180 ms) and at 410 nm (for 300 ms). The emission light was filtered with a 505-nm DRLP filter (Chroma Technologies). Acquisitions were performed every 1 s. Images were background subtracted and analyzed with ImageJ, calculating the ratio (R, proportional to [Ca^2+^]) between the emissions collected after excitation at 475 nm and 410 nm, respectively.

To evaluate ER–mitochondria Ca^2+^ transfer, for both cytosolic and mitochondrial Ca^2+^ measurements, an IP3-generating agonist solution containing 100 μM ATP + 300 μM carbachol + 100 μM glutamate was added when indicated in the graphs. The addition was performed in Ca^2+^-free, EGTA-containing solution.

### Synaptosome isolation and ATP level quantification

Mouse forebrain was homogenized in Syn-PER reagent (Thermo Fisher, Cat. no. 87793) (10 mL per gram of tissue) using a Dounce grinder on ice with 10 strokes and then centrifuged at 1200*g* for 10 min at 4 °C. The supernatant was isolated and centrifuged at 15,000*g* for 20 min at 4 °C to yield a pellet containing our crude synaptosomal preparation. Pellet was resuspended in 1 mL of Ca^2+^-containing HBSS and kept on ice until protein quantification was performed. Ten micrograms of synaptosomes per condition/replicate was pretreated with luteolin (2.5 μM) in the presence or absence of XeC (5 μM) for 30 min (30 °C) and briefly stimulated for 5 min with 30 mM KCl to keep the ER Ca^2+^ store filled [37]. ATP levels were quantified by luminescence measurement using the CellTiter-Glo assay.

### Individual mitochondrial complex activity

Mitochondria from mouse brain were isolated as previously described [36] and incubated with 2.5 μM luteolin or 0.1% DMSO for 30 min. 2.5 μg of isolated mitochondria diluted in mitochondrial assay solution (MAS: 70 mM sucrose, 220 mM mannitol, 10 mM K_2_HPO_4_, 5 mM MgCl_2_, 1 mM EGTA, 2 mM HEPES–KOH, pH 7.2) supplemented with 0.2% (w/v) fatty acid-free BSA, 10 mM pyruvate, 2 mM malate, and 4 μM FCCP was seeded into poly(ethylenimine)-coated (1:15,000; Sigma-Aldrich, catalog no: 03880) XF96 Seahorse plates by centrifugation at 2000*g* for 18 min, at 4 °C [36, 75]. After centrifugation, Seahorse plates were equilibrated in a humidified CO_2_-free incubator at 37 °C for 10–15 min. Electron flow through the electron transport chain was evaluated after sequential injection of Rot (2 μM; complex I inhibitor), succinate (10 mM; complex II substrate), Ant A (4 μM; complex III inhibitor), and ascorbate/TMPD (N,N,N′,N′-tetramethyl p-phenylenediamine) (10 mM/100 μM electron donors to cytochrome C/complex IV).

### *C. elegans* strains, maintenance, and trashing assay

Nematodes were maintained at 20 °C following standard culture methods. The following strain was used: AM101 *rmIs110[F25B3.3p::Q40::YFP]*. Nematodes were grown on nematode growth medium (NGM) plates on an *Escherichia coli* OP50 lawn. For treatments, DMSO and luteolin were added at 25 μM (final concentration) in liquid NGM before pouring into sterile plastic petri dishes. For the trashing assay, gravid adult nematodes were synchronized by hypochloride treatment. At day 4 from hatching, nematodes were transferred to DMSO- and luteolin-containing NGM plates. After 96 h, single nematode was transferred into one drop of M9 buffer (3 g KH_2_PO_4_, 6 g Na_2_HPO_4_, 5 g NaCl, 1 ml 1 M MgSO_4_, water to 1 l) and the rhythmic bending was assessed over 90 s. Each experiment was conducted in triplicate.

### Statistical analysis

Results are expressed as mean ± SEM (standard error of the mean) of the number of independent experiments or animals used. The number of experiments per experimental group/condition is indicated in the legends when individual points are not represented in the graphs. Comparisons between multiple groups were performed by non-parametric one-way analysis of variance (ANOVA) using Kruskal-Wallis test. Correction for multiple comparisons was done by two-way ANOVA followed by Tukey or Sidak multiple comparison test. Comparison between two groups was performed by non-parametric Mann-Whitney test. The *F*-test was performed to analyze the interaction term. Significance was accepted at *p* < 0.05. Outliers were identified using the ROUT method (*Q* = 1%). Only outliers were eliminated. All analyses were performed using Prism software (GraphPad Version 8.0).

## Supplementary Information

**Additional file 1:** **Figure S1.** Characterization of differentiated SH-SY5Y cells. **Figure S2.** Cytotoxicity of compounds evaluated in the HTS. **Figure S3.** Effects of luteolin in respiratory capacity and ΔΨ_m_. **Figure S4.** Effects of luteolin in mitochondrial biogenesis and cristae organization. **Figure S5.** Effects of luteolin on calcium levels and characterization of isolated mitochondrial fractions.

**Additional file 2.** Prestwick Chemical Library compounds used in the HTS and the corresponding ATP and cytotoxicity z-scores (data related with Fig. 2b and Additional file 1: Figure S2A).

**Additional file 3.** ATP and cytotoxicity z-scores obtained for the 3-CRC (data related with Fig. 2c and Additional file 1: Figure S2D).

## Data Availability

All data generated or analyzed during this study are included in this article and its additional files. Supporting data values for Fig. 2 are provided as Additional files 2 and 3. Plasmids used in this research may be available upon request under MTA.

## Abbreviations

AKT: Protein kinase B
AMPK: 5′ AMP-activated protein kinase
Ant A: Antimycin A
ARE: Antioxidant responsive element
ATP5A: ATP synthase subunit α
BDNF: Brain-derived neurotrophic factor
CREB: Cyclic AMP response element-binding protein
CRC: Concentration response curve
ER: Endoplasmic reticulum
FDA: U.S. Food and Drug Administration
FBS: Fetal bovine serum
GRP75: Glucose-regulated protein 75
HD: Huntington’s disease
HTS: High-throughput screening
H_2_O_2_: Hydrogen peroxide
IP_3_R: Inositol 1,4,5-triphosphate receptor
Lut: Luteolin
MAM: Mitochondria-associated endoplasmic reticulum membrane
MAP2: Microtubule-associated protein 2
MCU: Mitochondrial calcium uniporter
mtDNA: Mitochondrial DNA
MERCS: Mitochondria-endoplasmic reticulum contact sites
mHTT/mHtt: Human/non-human mutant huntingtin
OCR: Oxygen consumption rate
OxPHOS: Oxidative phosphorylation
NADH: Nicotinamide adenine dinucleotide (reduced form)
Nrf2: Nuclear factor erythroid 2-related factor 2
NRF1/2: Nuclear-derived transcription factors 1/2
PDH: Pyruvate dehydrogenase
PGC-1α: Peroxisome proliferator-activated receptor-γ coactivator
PSD95: Postsynaptic density protein 95
RA: Retinoic acid
ROS: Reactive oxygen species
SERCA2a: SR/ER Ca^2+^-ATPase 2a
SNAP25: Synaptosome-associated protein 25
SPLICS: Split-GFP-based contact site sensor
SR: Sarcoplasmic reticulum
TEM: Transmission electron microscopy
TFAM: Transcription factor A, mitochondrial
TOM70: Translocase of the mitochondrial outer membrane 70
YAC: Yeast artificial chromosome
UCP2: Uncoupling protein 2
VAPB: Vesicle-associated membrane protein-associated protein B/C
VDAC1: Voltage dependent anion channel 1
XeC: Xestospongin C
ΔΨ_m_: Mitochondrial membrane potential
