# Supplementary material for: Neuronal cell-based high-throughput screen for enhancers of mitochondrial function reveals luteolin as a modulator of mitochondria-endoplasmic reticulum coupling
Source: BMC Biol. 2021 Mar 24;19:57. doi: 10.1186/s12915-021-00979-5 (PMC7989211; doi:10.1186/s12915-021-00979-5)
Supplement: Supplementary file 1 — Additional file 1: Figure S1. Characterization of differentiated SH-SY5Y cells. Figure S2. Cytotoxicity of compounds evaluated in the HTS. Figure S3. Effects of luteolin in respiratory capacity and ΔΨm. Figure S4. Effects of luteolin in mitochondrial biogenesis and cristae organization. Figure S5. Effects of luteolin on calcium levels and characterization of isolated mitochondrial fractions. [file 12915_2021_979_MOESM1_ESM.docx]

**Neuronal cell-based high throughput screen reveals luteolin as mitochondrial enhancer through modulation of mitochondria-endoplasmic reticulum coupling**

Luana Naia, Catarina M. Pinho, *et al.*

**Additional file 1**

**
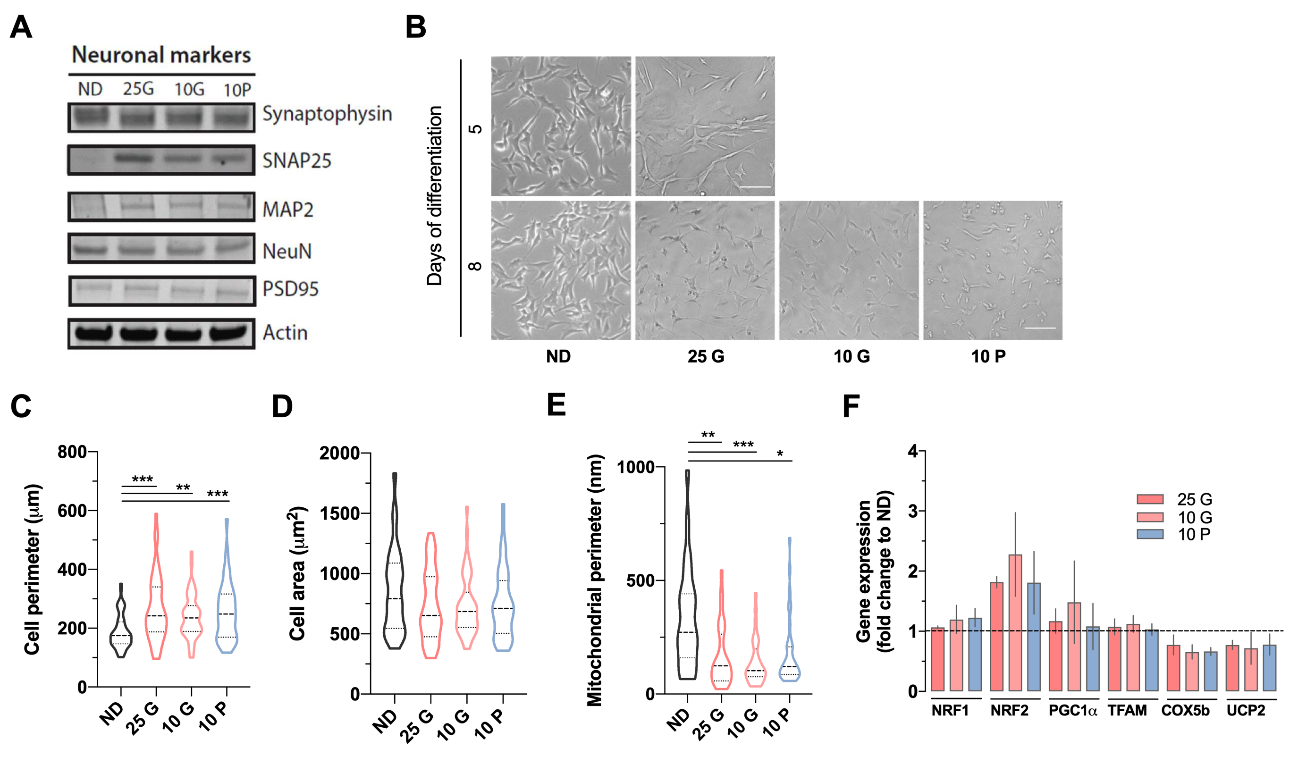
**

**Additional file 1: Figure S1 – Characterization of differentiated SH-SY5Y cells grown in glucose- or pyruvate-enriched media.**

**(A)** Synaptic markers were evaluated by western blotting using specific antibodies for synaptophysin, SNAP25, MAP2, NeuN and PSD95. Actin was used as protein loading control.

**(B-D)** Images of cultured cells were taken after 5 and 8 days *in vitro* using different differentiation protocols. Perimeter and cellular area and were quantified using ImageJ (n=28 ND, n=32 25 G, n=30 10 G, n=34 10 P from 4 independent experiments). ND = non-differentiated; 25 G = 25 mM glucose; 10 G = 10 mM glucose; 10 P = 10 mM pyruvate. Scale bar = 100 μm.

**(E)** Mitochondrial perimeter (in nm) per mitochondria profile was quantified in electron micrographs from SH-SY5Y (n=20 for all conditions from 4 independent experiments).

**(F)** Gene expression analysis of mitochondrial-related transcription factors and mitochondrial complex subunits were quantified by qPCR (3 independent experiments run in triplicates).

**Statistical significance:** ^*^p<0.05, ^**^p<0.01, ^***^p<0.001 using non-parametric Kruskal-Wallis test.


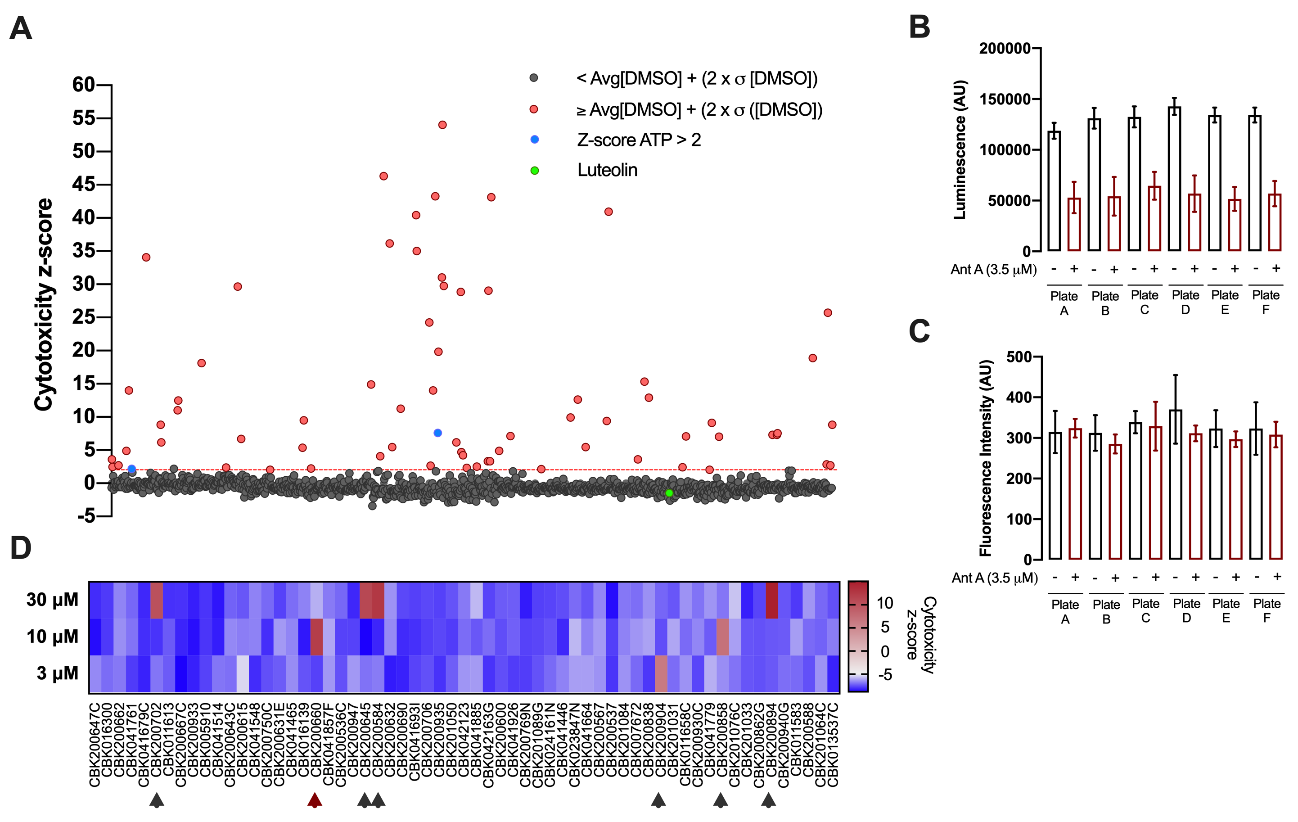


**Additional file 1: Figure S2 – Cytotoxicity of compounds evaluated in the HTS.**

**(A)** 10 P-differentiated SH-SY5Y cells were incubated with 1,200 Prestwick library-selected compounds (10 μM, 24 h), which were automatically dispensed in 384-multiwells plates. Cytotoxicity was evaluated by fluorescence, and compounds were considered toxic when z-score were higher than 2 (red dots). Compounds with ATP z-score higher than 2 that were validated as toxic are displayed in blue. Luteolin is highlighted in green.

**(B, C)** In each plate of the HTS, Ant A was used as a negative control for ATP levels displayed in B. No effects were observed in cytotoxicity (evaluated by fluorescence in C) validating Ant A as a mitochondrial toxin.

**(D)** Selected hits with ATP z-score ≥ 2 in Fig. 2B were tested for cytotoxicity in a 3-DRC. Toxic compounds are indicated by arrowheads. Burgundy arrowhead indicates a toxic compound with ATP z-score ≥ 2 for at least one tested concentration.

**
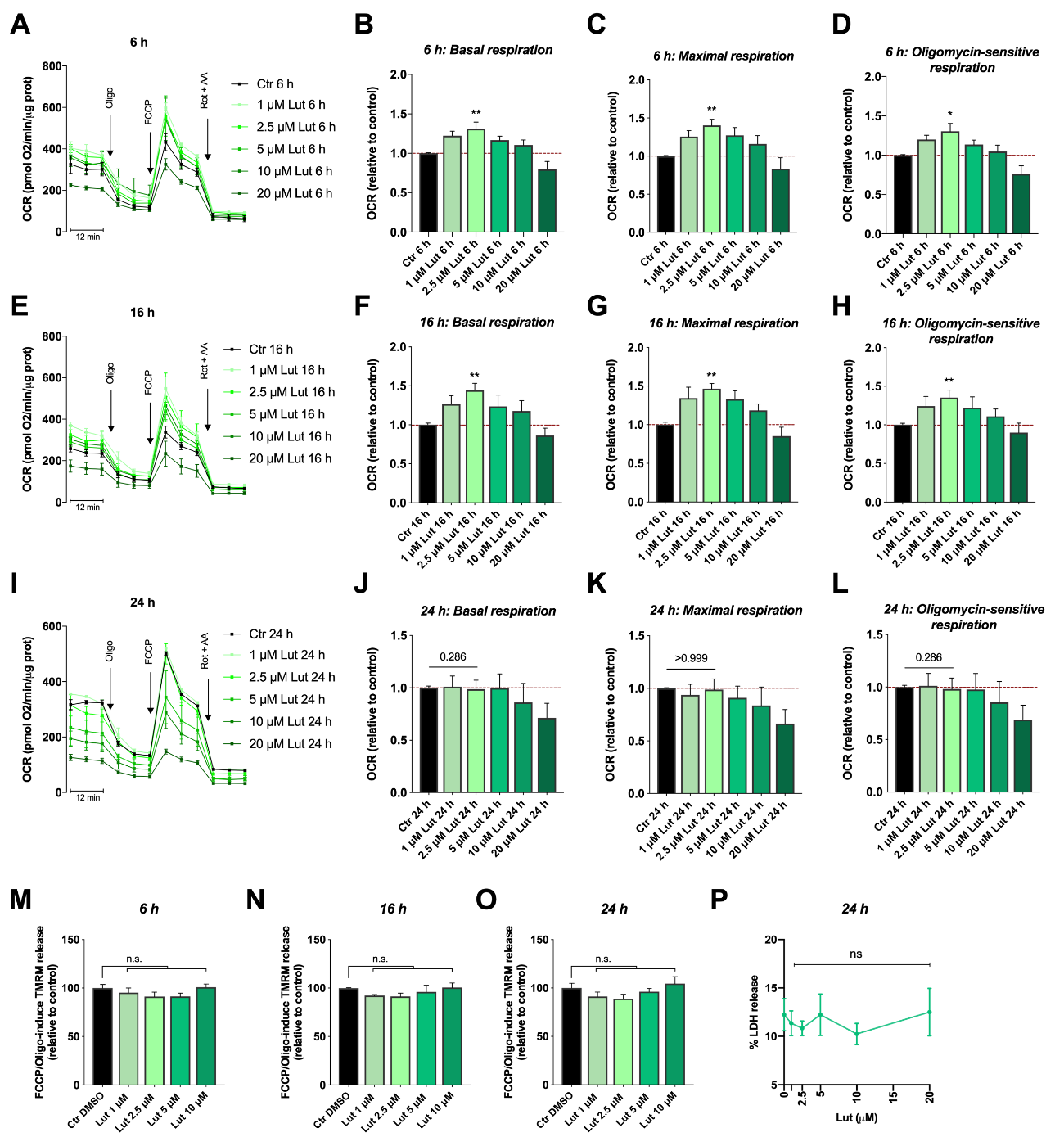
**

**Additional file 1: Figure S3 – Luteolin increases mitochondrial respiratory capacity at 6 h and 16 h of incubation.** Luteolin was added to cortical neurons (at indicated concentrations) for 6 h (**A-D, M**), 16 h (**E-H, N**) and 24 h (**I-L, O, P**) before experiments.

(**A-L**) OCR was measured using Seahorse analyzer (n=6-7 run in triplicate).

**(M-O)** Mitochondrial membrane potential was evaluated using TMRM in quenching conditions (150 nm; 30 min) after mitochondrial depolarization with FCCP (2.5 µM) plus oligomycin (2 µg/mL) (n=4 run in triplicate).

**(P)** LDH release assay was performed to evaluate cytotoxicity (n=4 run in triplicate).

**Statistical significance:** ^*^p<0.05, ^**^p<0.01, using non-parametric Kruskal-Wallis test; n.s. = non-significant.


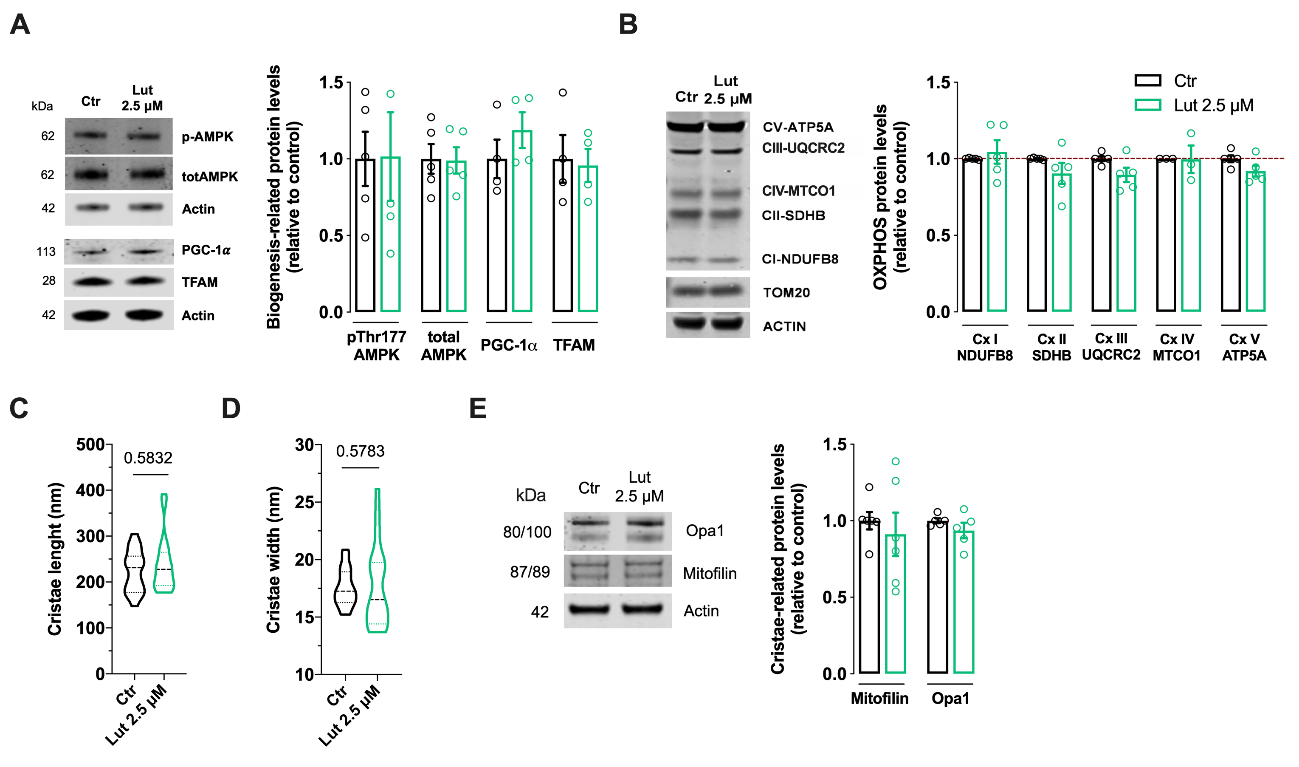


**Additional file 1: Figure S4 – Luteolin effect on protein expression of OxPHOS subunits and regulators of cristae organization and mitochondrial biogenesis.**

**(A, B, E)** Primary neurons were treated with DMSO or Luteolin (2.5 μM) for 16 and expression of several proteins were quantified by Western blotting using specific antibodies described in Material and Methods (n=4-6; each point in the graph represents one independent experiment).

**(C, D)** Cristae length and width (in nm) were quantified by TEM (n=19 from 3 independent experiments).

**
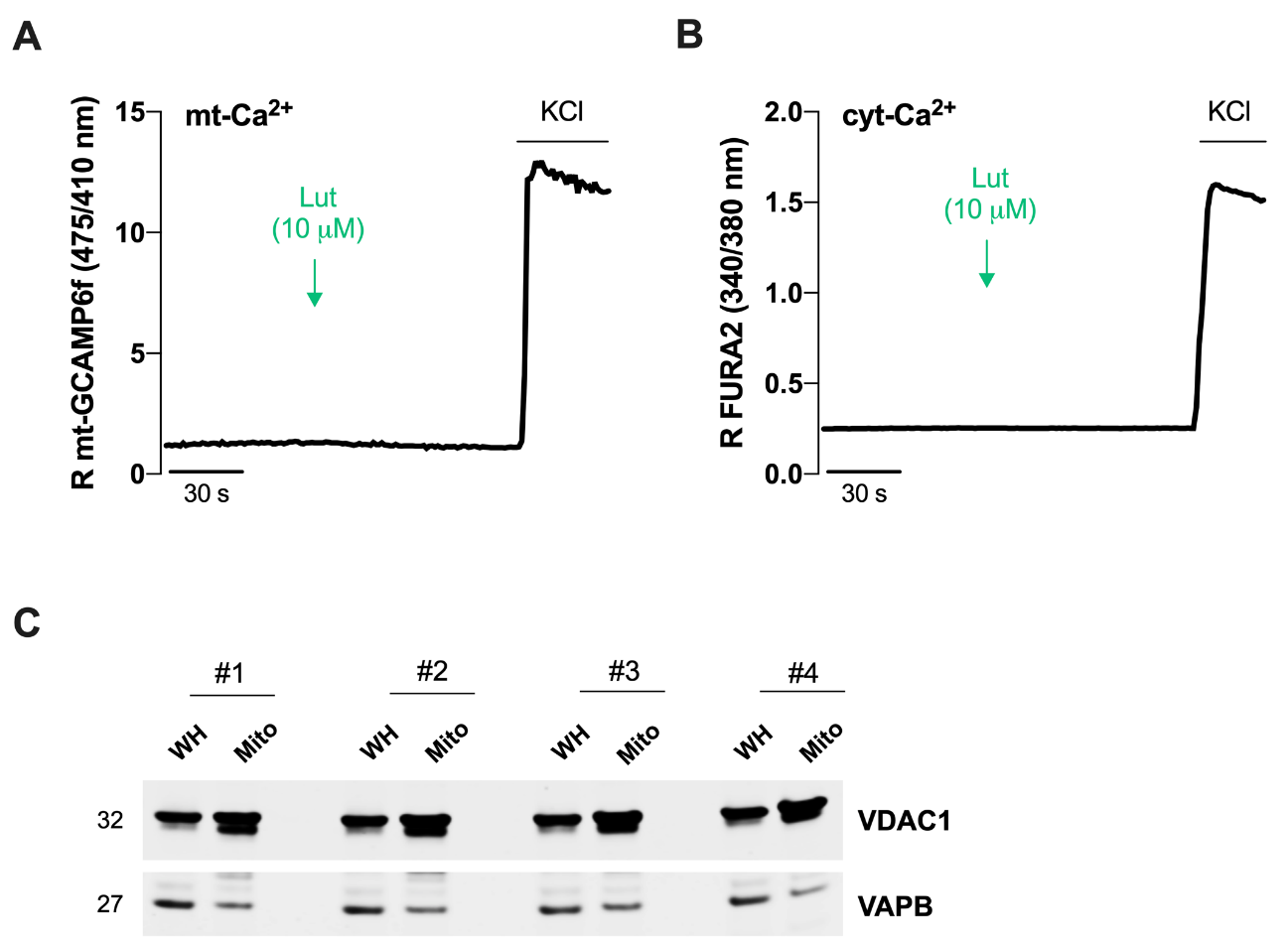
**

**Additional file 1: Figure S5 – Evaluation of acute luteolin stimulation on mitochondrial and cytosolic Ca^2+^ signaling and characterization of isolated mitochondrial fractions.**

**(A, B)** Representative traces of mitochondrial (mt-Ca^2+^, A) and cytosolic (cyt-Ca^2+^, B) Ca^2+^ levels in cortical neurons. The arrow indicates the addition of luteolin (10 μM) to understand if this flavonoid could induce an acute Ca^2+^ response in the two compartments. At the end of the experiment 100 mM KCl was added.

**(C)** Samples from four different mitochondrial preparation used in the Fig. 4 H, I, were run in a western blot. Isolated mitochondria (Mito) show remarkably increased VDAC1 staining in comparison with the whole homogenate (WH). VAPB staining confirm the presence of mitochondria-associated ER membranes in the preparation.
